# Frequent Long-Range Epigenetic Silencing of Protocadherin Gene Clusters on Chromosome 5q31 in Wilms' Tumor

**DOI:** 10.1371/journal.pgen.1000745

**Published:** 2009-11-26

**Authors:** Anthony R. Dallosso, Anne L. Hancock, Marianna Szemes, Kim Moorwood, Laxmi Chilukamarri, Hsin-Hao Tsai, Abby Sarkar, Jonathan Barasch, Raisa Vuononvirta, Chris Jones, Kathy Pritchard-Jones, Brigitte Royer-Pokora, Sean Bong Lee, Ceris Owen, Sally Malik, Yi Feng, Marcus Frank, Andrew Ward, Keith W. Brown, Karim Malik

**Affiliations:** 1 Cancer and Leukaemia in Childhood – Sargent Research Unit, Department of Cellular and Molecular Medicine, School of Medical Sciences, University of Bristol, Bristol, United Kingdom; 2 Department of Biology and Biochemistry, University of Bath, Bath, United Kingdom; 3 Department of Medicine, Columbia University College of Physicians and Surgeons, New York, New York, United States of America; 4 Paediatric Oncology, Institute of Cancer Research/Royal Marsden NHS Trust, Sutton, United Kingdom; 5 Institute of Human Genetics and Anthropology, Heinrich-Heine University of Duesseldorf, Duesseldorf, Germany; 6 Genetics of Development and Disease Branch, National Institute of Diabetes and Digestive and Kidney Diseases, National Institutes of Health, Bethesda, Maryland, United States of America; 7 Department of Biochemistry, School of Medical Sciences, University of Bristol, Bristol, United Kingdom; 8 Developmental Biology, Institute of Biology 1, University of Freiburg, Freiburg, Germany; Medical Research Council Human Genetics Unit, United Kingdom

## Abstract

Wilms' tumour (WT) is a pediatric tumor of the kidney that arises via failure of the fetal developmental program. The absence of identifiable mutations in the majority of WTs suggests the frequent involvement of epigenetic aberrations in WT. We therefore conducted a genome-wide analysis of promoter hypermethylation in WTs and identified hypermethylation at chromosome 5q31 spanning 800 kilobases (kb) and more than 50 genes. The methylated genes all belong to *α-*, *β-*, and *γ-protocadherin* (*PCDH*) gene clusters (Human Genome Organization nomenclature *PCDHA@*, *PCDHB@*, and *PCDHG@*, respectively). This demonstrates that long-range epigenetic silencing (LRES) occurs in developmental tumors as well as in adult tumors. Bisulfite polymerase chain reaction analysis showed that *PCDH* hypermethylation is a frequent event found in all Wilms' tumor subtypes. Hypermethylation is concordant with reduced *PCDH* expression in tumors. WT precursor lesions showed no *PCDH* hypermethylation, suggesting that de novo *PCDH* hypermethylation occurs during malignant progression. Discrete boundaries of the *PCDH* domain are delimited by abrupt changes in histone modifications; unmethylated genes flanking the LRES are associated with permissive marks which are absent from methylated genes within the domain. Silenced genes are marked with non-permissive histone 3 lysine 9 dimethylation. Expression analysis of embryonic murine kidney and differentiating rat metanephric mesenchymal cells demonstrates that *Pcdh* expression is developmentally regulated and that *Pcdhg@* genes are expressed in blastemal cells. Importantly, we show that PCDHs negatively regulate canonical Wnt signalling, as short-interfering RNA–induced reduction of *PCDHG@* encoded proteins leads to elevated β-catenin protein, increased β-catenin/T-cell factor (TCF) reporter activity, and induction of Wnt target genes. Conversely, over-expression of PCDHs suppresses β-catenin/TCF-reporter activity and also inhibits colony formation and growth of cancer cells in soft agar. Thus PCDHs are candidate tumor suppressors that modulate regulatory pathways critical in development and disease, such as canonical Wnt signaling.

## Author Summary

The development of tissues and organs in the human body requires carefully regulated production of proteins by cells. Proteins permit the growth and development of the many varied structures required for a healthy body. In many diseases, including some cancers, tissues and organs fail to develop as they should due to the normal production of proteins being changed. The work presented here shows that in Wilms' tumor, a childhood cancer of the kidney, a large group of related proteins that are likely necessary for growth and development of a normal kidney are not produced properly. This is due to their production being switched off within the cancer cells. We show how these proteins, known as protocadherins, can themselves alter the function of other proteins already known to be important in normal growth and cancer. Thus our study increases our understanding of how protocadherins are important in normal growth and of how altering protocadherins may lead to disease, such as cancer.

## Introduction

Wilms' tumour (WT) represents a paradigm for cancer arising from disrupted development. Failure of the metanephric blastemal cells to undergo mesenchymal to epithelial transition, together with proliferation of these undifferentiated cells, is intrinsic to the development of Wilms' tumours [1]. Thus WT predisposing genes are often critical in normal nephrogenesis. As the aetiology of WTs cannot be explained solely by the known genetic changes, we have evaluated epigenetic changes in WTs. Several epigenetic lesions have previously been identified in Wilms' tumour, in particular loss of imprinting at chromosome 11p13 [2],[3] and 11p15 [4], which we have shown to be early and independent events [5]. In common with other cancers, WTs also show tumour suppressor gene silencing which includes genes such as *HACE1* (73% of tumours analysed) [6], *RASSF1* (56%) [7], *CASP8* (43%), *MGMT* (30%), *RASSF5/NORE1* (15%), and *CDKN2A* (10–15%) [8]. In addition, we have recently shown over-expression of the *GLIPR1* gene resulting from promoter hypomethylation (87%) [9].

In order to identify candidate genes involved in Wilms' tumorigenesis, we undertook genome-wide analysis of promoter methylation. We have identified pronounced tumour-specific hypermethylation in a region spanning ∼800 kb of chromosome 5q31. This region contains members of the *PCDH* superfamily in 3 multi-gene clusters (*PCDHA@, PCDHB@ and PCDHG@*) [10]. Hypermethylation of this locus is an example of long range epigenetic silencing, which has previously been reported in colorectal cancer at chromosome 2q14.2 [11], the *MLH1* locus on 3p22 [12], and for the *HOXA* gene cluster on chromosome 7p15 in breast and lung cancers [13],[14]. We demonstrate that silencing of gene expression is concomitant with DNA hypermethylation and repressive histone modifications. Although little is known about the functions of these clustered PCDHs, other members of the PCDH superfamily have been shown to have tumour suppressor activity, such as PCDH10 in various carcinomas [15],[16] and PCDH8 in breast cancer [17]. Functional data presented here suggest that proteins encoded by the chromosome 5q31 PCDHs modulate the Wnt pathway and are candidate Wilms' tumour suppressor genes.

## Results

### A large hypermethylated domain at 5q31 in Wilms' tumour

Following methylated DNA immunoprecipitation (MeDIP, Figure S1A), the efficacy and specificity of the MeDIP protocol was verified using quantitative real-time polymerase chain reaction (PCR) of a constitutively methylated CpG island (CGI) at the *H19* imprinting control region, demonstrating successful enrichment relative to input DNA. Specificity for methylated CGIs was shown by PCR of the *RASSF1* 5′ CGI. Amplification of a non-CGI sequence (an intragenic region of the *TBP* gene), was used as a negative control for methylated DNA enrichment (Figure S1B). Validated MeDIP DNA samples from human fetal kidney and Wilms' tumours were then hybridised to Nimblegen HG18 Refseq promoter microarrays (MeDIP-chip) to detect tumour-specific alterations in methylation.

Microarray data from 5 sporadic WTs were examined to identify promoters displaying tumour-specific hypermethylation (*P*<0.05, *t*-test). Overall we found 2043 hypermethylated genes. Significantly, a large subset of these targets were found in 2 or more tumours; pairwise comparisons showed that each individual tumour had between 12.0% and 33.7% (mean 23.8%) of hypermethylated targets in common with the other tumours analysed. The genomic distribution of recurrently hypermethylated genes was non-random (*P*<0.001, chi square test), with many being located together or within gene clusters (Table S1). Contiguous hypermethylated genes were more likely to be found in several tumours (13 out of 17 loci).

**Figure 1:**
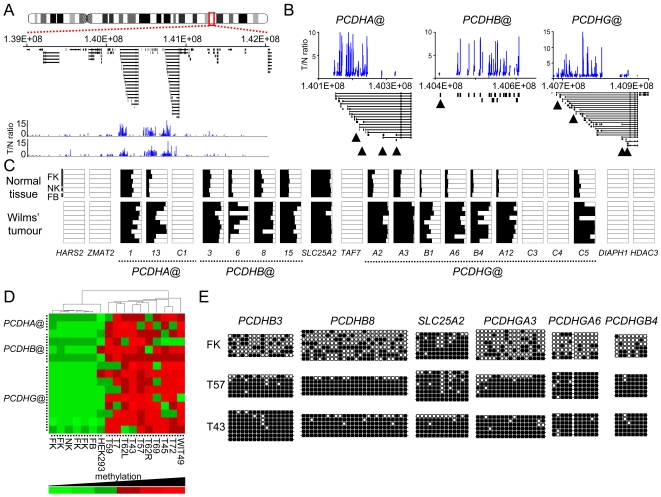
A large hypermethylated domain on chromosome 5q31 in Wilms' tumour. (A) The MeDIP-chip tumour/normal (T/N) signal ratio shown for 2 representative WTs identifies hypermethylation of multiple gene promoters across a domain spanning 800 kb on chromosome 5q31.3 (red box). (B) The MeDIP-chip profile of *PCDHA@*, *PCDHB@* and *PCDHG@* in a representative WT. A minority of genes escaping hypermethylation are indicated (black arrowheads). (C) DNA methylation assayed using COBRA. Percentage methylation was analysed by gel densitometry for each gene, and is represented by horizontal bars (black  =  percentage methylated, white  =  percentage unmethylated). Data from 6 normal tissues (4 fetal kidneys [FK], postnatal kidney [NK] and fetal brain [FB]) and 9 Wilms' tumours are shown. Non-*PCDH* genes flanking the domain and between *PCDHB@* and *PCDHG@* are also shown. (D) Unsupervised hierarchical clustering of COBRA methylation data from normal and tumour tissues (T), HEK293 and WiT49 cell lines. (E) Bisulfite sequencing of *PCDHB3*, *PCDHB8*, *SLC25A2*, *PCDHGA3*, *PCDHGA6* and *PCDHGB4 5′* CGIs in normal fetal kidney (FK) and 2 Wilms' tumours, T43 & T57.

Strikingly, of 146 annotated genes hypermethylated in 3 or more tumours, 25 were *PCDHs* located at chromosome 5q31 and MeDIP-chip data revealed enriched tumour/normal ratios across 50 closely-linked CGIs spanning 800 kb on chromosome 5q31.3 (Figure 1A and Figure S1C). Hypermethylation at this locus was identified in all tumours examined (Table S2) and hypermethylated CGIs localise to *PCDHA@, PCDHB@ and PCDHG@*. Each *PCDHB@* gene is encoded by a single, unspliced exon, while the *PCDHA@* and *PCDHG@* genes have unique 5′ exons splicing into cluster-specific constant region exons encoding invariant cytoplasmic domains [10]. Proximal and distal boundaries to the hypermethylated domain were apparent (Figure 1A) and, remarkably, tumour MeDIP signal was enriched at virtually all of the *PCDH* CGIs across the locus (Figure 1B). As a complementary screening approach, we also conducted enrichment of methylated DNA from the WiT49 Wilms' tumour cell-line [18] using recombinant methyl-CpG binding domain (MBD) protein, which permitted microarray methylation profiling without probe DNA amplification. This analysis also showed hypermethylation of multiple *PCDHs* across the region (data not shown). Both genome-wide analyses identified genes previously known to be hypermethylated in Wilms' tumour, such as *RASSF1* (Table S2). Thus our genome-wide promoter analysis identifies a novel region of long-range epigenetic silencing in WTs on chromosome 5q31.3.

### Long-range epigenetic modifications at 5q31 are common in Wilms' tumour but not in preneoplastic lesions

We used combined bisulfite and restriction analysis (COBRA) to validate and characterise methylation in normal and tumour DNAs. Methylation profiles were consistent with the microarray data, and locus-wide hypermethylation was observed in all tumours and WiT49 cells (Figure 1C and 1D). We also analysed an additional thirteen *PCDHs* that were identified as hypermethylated by MeDIP-chip, demonstrating that hypermethylation varied between individual tumours (Figure 1C and 1D). Of 19 *PCDHG@* genes, 15 were hypermethylated according to COBRA and array analysis, together with 15/16 *PCDHB@* genes and 13/15 *PCDHA@* genes. Bisulfite sequencing data was consistent with COBRA and MeDIP-chip data (Figure 1E). In total, we analysed 38 WTs (Table S2) and all display hypermethylation of multiple *PCDHs*, with many *PCDHs* showing a very high frequency of hypermethylation in tumours. These include *PCDHGA3*, hypermethylated in 24/27 tumours (89%), *PCDHGB4* (23/25, 92%), *PCDHGA2* (11/13, 85%) and *PCDHB3* (21/33, 64%). COBRA analyses of *PCDHAC1*, *PCDHAC2, PCDHB1, PCDHGC3* and *PCDHGC4* showed no evidence of hypermethylation, consistent with MeDIP-chip data (Figure 1B and 1C and Table S2).

Analysis of *PCDH* methylation in microdissected perilobar nephrogenic rests, presumptive WT precursor lesions, revealed no *PCDH* hypermethylation (Figure S3) but hypermethylation was evident at the *PCDHGA3* and *PCDHGB4* genes in a set of stromal-predominant tumours (Figure S4). Taken together our data show that hypermethylation occurs at high frequencies in all WT subtypes, and strongly suggests that *de novo* methylation arises during neoplastic progression from nephrogenic rest to Wilms' tumour.

There was no tumour-specific hypermethylation apparent at upstream and downstream CGIs within 100 kb (eight promoter CGIs in total) of the *PCDH* clusters. A non-clustered protocadherin gene on chromosome 5q31, *PCDH1*, 300 kb telomeric to *PCDHG@*, was also unmethylated. Two non-*PCDH* genes are situated within the methylated domain; *SLC25A2* was constitutively methylated in both normal tissues and tumours and *TAF7* was constitutively unmethylated (Figure 1C and Figure S2). Thus, their methylation is unaffected by the surrounding epigenetic defect, and tumour-specific hypermethylation is specific to clustered *PCDH*s.

### Silencing of *PCDH* expression in Wilms' tumour

**Figure 2:**
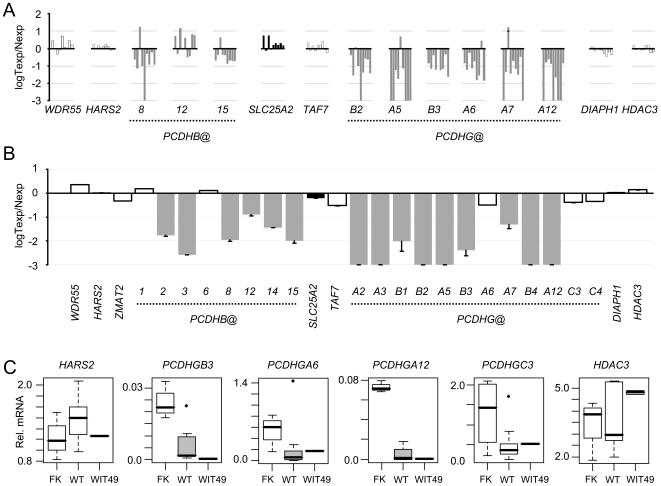
Silencing of *PCDH* expression in Wilms' tumour. (A) Expression levels of genes across the locus in 9 tumours (Texp), relative to the mean of 4 normal fetal kidney samples (N exp). Grey bars are used for genes showing tumour-specific hypermethylation, white bars for unmethylated genes and black bars for the constitutively methylated *SLC25A2* gene. (B) Expression levels of 5q31 transcripts correspond with DNA methylation status in WiT49 cells. Expression of unmethylated genes (white bars), constitutively methylated genes (black bar) and hypermethylated *PCDH*s (grey bars) are shown. (C) Suppression of methylated and unmethylated *PCDH*s within the chromosome 5q31 LRES. Gene expression levels relative to the house-keeping gene *TBP* are shown. Horizontal black line, median value; box, interquartile range; whiskers, data range excluding outliers; black dots, outliers (defined as those data points greater than range multiplied by inter-quartile range beyond the box). Grey boxes give samples shown to be hypermethylated, open boxes represent unmethylated samples.

Quantitative RT-PCR showed negligible *PCDHA@* expression in kidney. However, *PCDHB@* and *PCDHG@* transcripts were readily detectable in fetal kidney, and consistent suppression of methylated *PCDHG@* transcripts was apparent in tumours relative to kidney. Of 11 hypermethylated *PCDHG@* genes analysed in our tumour panel, 9 showed greater than 90% repression, with the remaining 2 showing very low basal expression in fetal kidney. For many *PCDHs*, expression in tumours was decreased over 100-fold or below the limits of detection (Figure 2A). *PCDHGC3* was consistently unmethylated in WTs (Figure 1C), but *PCDHGC3* expression was lowered in WTs relative to normal kidney (Figure 2C). Interestingly, WiT49 *PCDH* methylation and expression reflects the general silencing pattern observed with WTs (Figure 2B), but in addition to *PCDHGC3, PCDHGA6* is also unmethylated in WiT49 cells. Despite the absence of hypermethylation, reduced expression of both *PCDHGA6* and *PCDHGC3* transcripts relative to fetal kidney was apparent in Wit49 cells. Expression of *PCDHGA6* was, however, further suppressed in WTs with *PCDHGA6* hypermethylation (Figure 2C).

Expression of *PCDHB@* genes in tumours was more variable than the *PCDHG@* genes. *PCDHB8* and *PCDHB15* exhibited strong methylation associated silencing but *PCDHB12* was not consistently down-regulated in tumours, despite hypermethylation (Figure 2A). All seven methylated *PCDHB@* genes analysed were, however, concordantly silenced in WiT49 cells (Figure 2B). Unmethylated genes outside the LRES boundary, such as *HARS2* and *HDAC3*, were not suppressed (Figure 2A and 2C).

To further establish the relationship between methylation and expression, we treated WiT49 cells with 5-azacytidine, which induced the expression of epigenetically silenced *PCDHB@* and *PCDHG@* genes (Figure S5); in contrast, genes located outside the hypermethylated domain, which showed unaltered expression in tumours (Figure 2), were unaffected by 5-azacytidine. This substantiates the mechanistic link between methylation levels and gene expression at this locus.

In summary, our expression analyses demonstrate that *PCDHB@* and *PCDHG@* expression occurs in human kidney, and that epigenetic silencing of gene expression occurs in WT.

### Profiling of histone modifications at active and silenced genes within the 5q31 LRES

**Figure 3:**
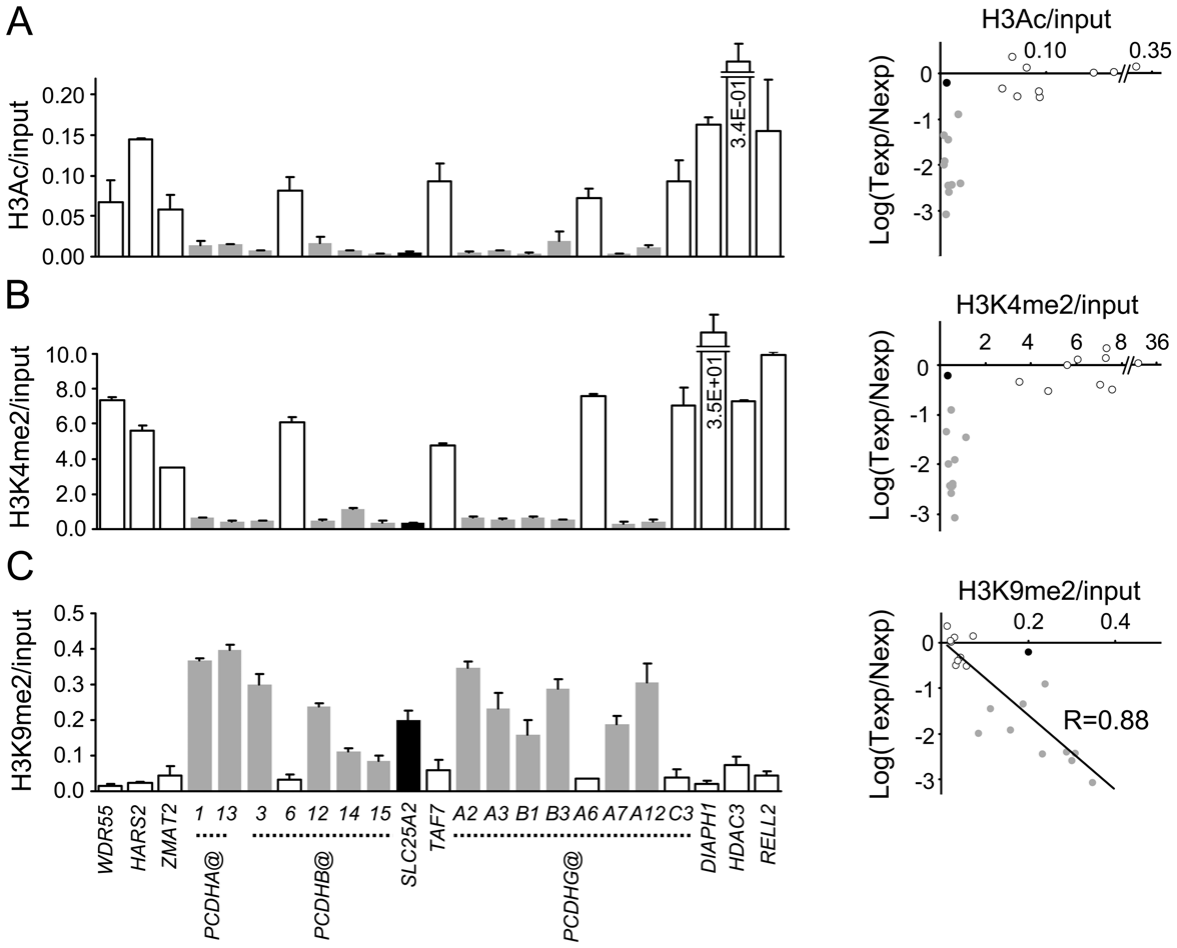
Hypermethylation across the chromosome 5q31 LRES is associated with specific histone modifications. Bar charts (left) show ChIP–quantitative PCR measuring relative levels of specific histone modifications at individual gene loci across the 5q31 locus in WiT49 cells. (A) H3Ac ChIP, (B) H3K4me2 ChIP, (C) H3K9me2 ChIP, all expressed relative to input DNA. Scatter plots (right) show the relationship between relative gene expression levels and histone modifications at each gene. The x-axis shows specific histone levels relative to input DNA, and the y-axis shows mRNA expression in WiT49 cells (Texp) relative to average mRNA expression in 4 fetal kidney samples (Nexp). Grey bars/datapoints signify genes hypermethylated in WiT49, open bars/datapoints show unmethylated genes, and the black bar/datapoint shows constitutively methylated *SLC25A2*.

We compared permissive (histone 3 dimethyl lysine 4, H3K4me2; histone 3 acetyl lysine, H3Ac) and repressive (histone 3 dimethyl lysine 9, H3K9me2) histone modifications at gene loci across the *PCDH* domain (Figure 3) by chromatin immunoprecipitation analysis (ChIP). Genes located outside the *PCDH* domain were enriched for H3K4me2 and H3Ac. Conversely, methylated *PCDH*s were enriched for H3K9me2. Hypermethylated *PCDHA@*, *PCDHB@* and *PCDHG@* genes all showed repressive histone modifications. As expected, the *SLC25A2* (constitutively methylated) and *TAF7* (unmethylated) genes were associated with repressive and permissive modifications, respectively. The *PCDHB6*, *PCDHGA6* and *PCDHGC3* genes, which were not hypermethylated in the WiT49 cell line, all had an active chromatin profile.

Comparison of gene expression levels, DNA methylation and histone marks (Figure 3, right panels) shows that DNA hypermethylation and silencing correlate with diminished H3Ac and H3K4me2, whereas “active” promoters have high H3Ac and H3K4me2 levels (Spearman rank order correlation coefficient, r = 0.67, *P* = 0.003, and r = 0.63, *P* = 0.006 respectively). H3K9me2 shows an opposite pattern, that is high levels at methylated/silenced genes (Spearman rank order correlation coefficient, r = −0.83, *P* = 0.0002). The degree of H3K9me2 enrichment displays proportionality to gene silencing (Pearson correlation coefficient, r = 0.88). Thus tumour-specific DNA hypermethylation is strongly linked with specific, repressive chromatin modifications, whereas unmethylated genes within, and flanking the region maintain an active chromatin configuration.

### PCDH expression in renal development and differentiation

**Figure 4:**
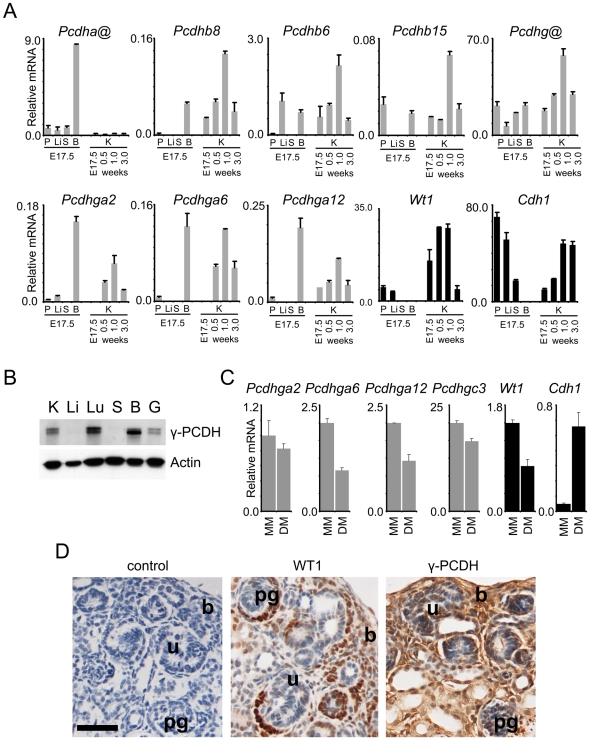
Developmental expression patterns of PCDHs. (A) *PCDH* transcript levels in mouse developmental tissues. Quantitative real-time expression analysis, relative to *Tbp*, in placenta (P), and E17.5 mouse fetal liver (Li), spleen (S), brain (B), and kidney (K) (E17.5, postnatal 0.5 week, 1 week, and 3 week) using assays specific for the constant region exons of *Pcdha@* and *Pcdhg@*, and individual *Pcdh* transcripts. Expression of *Wt1* and *Cdh1* are also shown (black bars). (B) Immunoblotting of human fetal tissue proteins with pan γ-PCDH antibody or actin for loading control. Samples are kidney (K), liver (Li), lung (Lu), spleen (S), brain (B), and gut (G). (C) Gene expression changes accompanying epithelial differentiation of rat metanephric mesenchyme following Lif, Fgf2, and Tgfα treatment. Quantitative real-time expression analysis, relative to *Tbp*, is shown for freshly dissected rat metanephric mesenchyme (MM) and differentiating mesenchyme (DM). (D) Immunohistochemical analysis of 1-day postnatal murine kidney with antibodies towards Wt1 and γ-PCDHs. Blastema (b), primitive glomeruli (pg), and ureteric buds (u) are labelled. The control panel shows a section where the primary antibody has been omitted. Bar  = 50 µm.

Previous studies have indicated that *PCDH* expression is largely restricted to neuronal tissues [10]. As we found abundant expression of *PCDHB@ and PCDHG@* genes in human fetal kidney (Figure 2), we examined *Pcdhb@ and Pcdhg@* expression during murine kidney development. Similar to humans, *Pcdha@* genes were expressed predominantly in brain, with negligible levels in kidney. In contrast, *Pcdhb@ and Pcdhg@* expression was abundant in kidney, with expression comparable to brain and exceeding placenta, liver and spleen. Transcript levels followed similar temporal profiles to *Wt1*, a mediator of mesenchymal-epithelial transition (Figure 4A). Postnatally, *Pcdh* expression decreases, in contrast to E-cadherin (*Cdh1*), where high expression levels are maintained in the mature organ. As our human expression analysis was restricted to comparing transcript levels in fetal kidney and Wilms' tumours (Figure 2), we also assessed other human fetal tissues for *PCDHG@* encoded proteins by immunoblotting in order to confirm abundant expression in fetal kidney. High expression was also evident in lung and brain, compared with moderate expression in gut. On longer exposure, low expression was also apparent in liver and spleen (Figure 4B).

In order to gain further insight on cell-type specific expression, we analysed *Pcdhb@* and *Pcdhg@* transcript levels during epithelial differentiation of rat metanephric mesenchymal cells in organ culture. This system has been shown to accurately reflect early differentiation in embryonic kidney [19]. Freshly isolated metanephric mesenchymal cells were found to express high *Pcdh* levels, and epithelial differentiation induced by growth factors was accompanied by down-regulation of *Pcdhga6*, *Pcdhga12* and *Wt1*. *Pcdhga2* and *Pcdhgc3* were also decreased upon differentiation, but to a lesser extent (Figure 4C). As expected, a sharp rise in *Cdh1* expression was observed, consistent with increasing epithelialisation.

Comparison of expression levels in murine developmental samples and rat mesenchymal cells suggests that the mesenchyme may be the principal cellular component expressing PCDHs in developing kidney. We therefore assessed expression of *Pcdhg@* encoded proteins (γ-PCDHs) immunohistochemically in postnatal day 1 murine kidney (Figure 4D). High expression was evident in the blastemal cells, with decreasing and more variable expression apparent in tubules and parietal epithelia. Subcellular staining was variable and included nuclear staining, which is consistent with a role for the γ-PCDH intracellular domain in gene regulation [20],[21]. Expression in the ureteric bud and visceral epithelia was low or absent. Thus γ-PCDHs proteins are evident in the murine equivalent of the presumptive multipotent cell of origin for WT. This is consistent with PCDHs having a role in kidney development and Wilms' tumorigenesis.

### Modulation of canonical Wnt signalling by PCDHs

Little is known about PCDH cellular functions, but a member of the PCDH superfamily, PCDH-PC, was previously shown to positively regulate β-catenin/TCF signalling [22]. The Wnt signalling pathway, which utilises the β-catenin/TCF transcriptional complex to programme developmental gene expression, is essential for nephrogenesis [23]. Constitutive Wnt signalling brought about by compromised β-catenin function is also involved in several cancers including WT [24]. We therefore assessed the possible effects of γ-PCDH knockdown on β-catenin/TCF mediated transcription using luciferase reporter plasmids which contain a minimal promoter adjacent to 7 tandem TCF binding sites (Super8xTOPFLASH, [25]). Short-interfering RNAs (siRNAs) were designed to target the constant region sequences of *PCDHG@* and transfected into WiT49 cells. Although WiT49 cells show extensive hypermethylation across the *PCDH* locus, specific *PCDH*s (for example, *PCDHGA6* and *PCDHGC3*) escape hypermethylation and are expressed, albeit at lower levels relative to fetal kidney (Figure 2C); WiT49 cells also exhibit intermediate Wnt signalling activity in the absence of β-catenin mutations, and are therefore suitable for investigating PCDH effects on the Wnt pathway.

**Figure 5:**
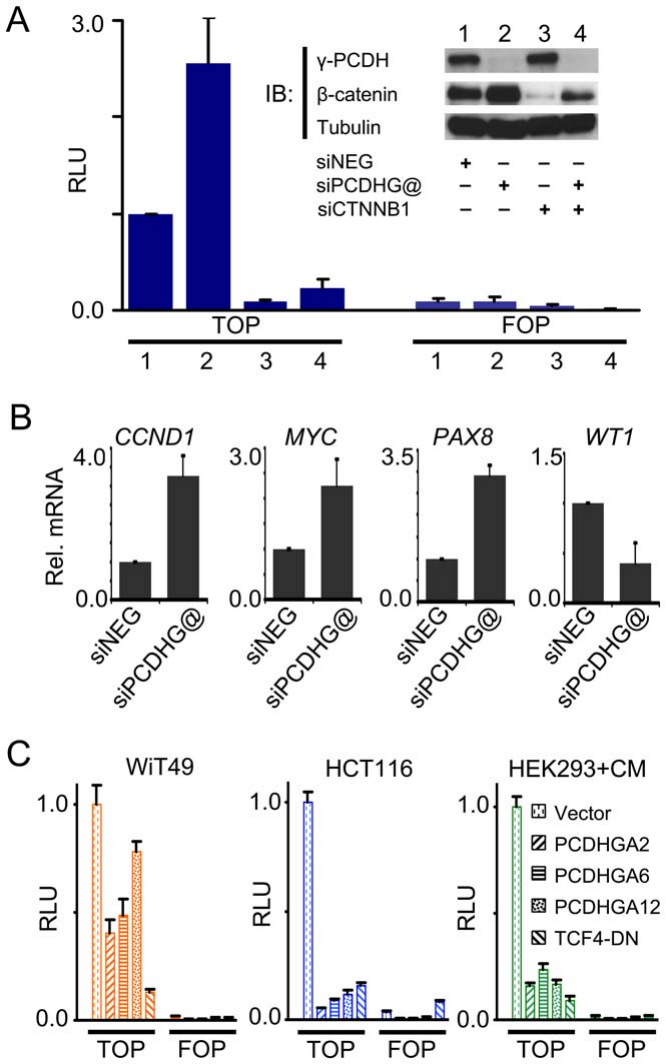
PCDH effects on Wnt signalling. (A) Enhanced β-catenin/TCF activity following γ-PCDH knockdown induced by *PCDHG@* constant region targeting siRNA, measured with Super8xTOPFLASH reporter (TOP). Super8xFOPFLASH (FOP) reporter is a negative control. RLU, relative luciferase units. Gamma-PCDH and β-catenin knockdowns are verified by immunoblotting (IB) in the inset, which also demonstrates increased cellular β-catenin accompanying γ-PCDH knockdown. (B) Quantitative real-time expression analysis, relative to *TBP*, showing induction of the Wnt pathway target genes *CCND1*, *CMYC*, and *PAX8* accompanying γ-PCDH knockdown; altered expression of *WT1* is also shown. (C) Repression of β-catenin/TCF reporter activity accompanying PCDH expression in WiT49, HCT116, and Wnt3a-conditioned medium treated HEK293 (HEK293+CM) cells. A plasmid containing a cDNA encoding dominant-negative TCF4 (TCF4-DN) is also shown as a positive control. Cells were co-transfected with PCDH expression vectors and Super8xTOPFLASH (TOP) or Super8xFOPFLASH (FOP) and luciferase activity measured. RLU, relative luciferase units.

The activity of the β-catenin/TCF reporter was increased by knockdown of γ-PCDHs, suggesting that γ-PCDHs negatively influence the canonical Wnt pathway. A second siRNA targeting a different sequence within the *PCDHG@* constant region gave similar results, negating the possibility of off-target effects (data not shown). Reporter upregulation was abolished by *CTNNB1* siRNA co-transfection, demonstrating that enhanced TCF-mediated activation was β-catenin-dependent (Figure 5A). This was further supported by immunoblot analysis which showed that knockdown of γ-PCDHs was accompanied by elevated β-catenin protein levels (Figure 5A, inset), although *CTNNB1* transcript levels were unchanged (data not shown). Knockdown of γ-PCDHs also induced expression of known β-catenin/TCF target genes *CCND1*, *CMYC* and *PAX8*, as well as repressing transcription of *WT1* (Figure 5B). We also individually over-expressed PCDHGA2, PCDHGA6 and PCDHGA12 in WiT49 cells, human embryonic kidney 293 cells (HEK293) treated with Wnt3a conditioned media and HCT116 cells to evaluate effects on β-catenin/TCF reporter activity (Figure 5C). The latter cell-line is derived from a colorectal cancer with an activating β-catenin mutation [26],[27] and also displays *PCDH* hypermethylation (Figure S6). PCDH-mediated suppression of β-catenin/TCF reporter activity was evident in all cell-lines, especially Wnt3a-treated HEK293 and HCT116, both of which have high Wnt signalling. As expected, expression of a dominant-negative form of TCF4 also strongly reduced reporter activity. Taken together, our data implicate *PCDHG@* encoded proteins in negative modulation of canonical Wnt signalling.

**Figure 6:**
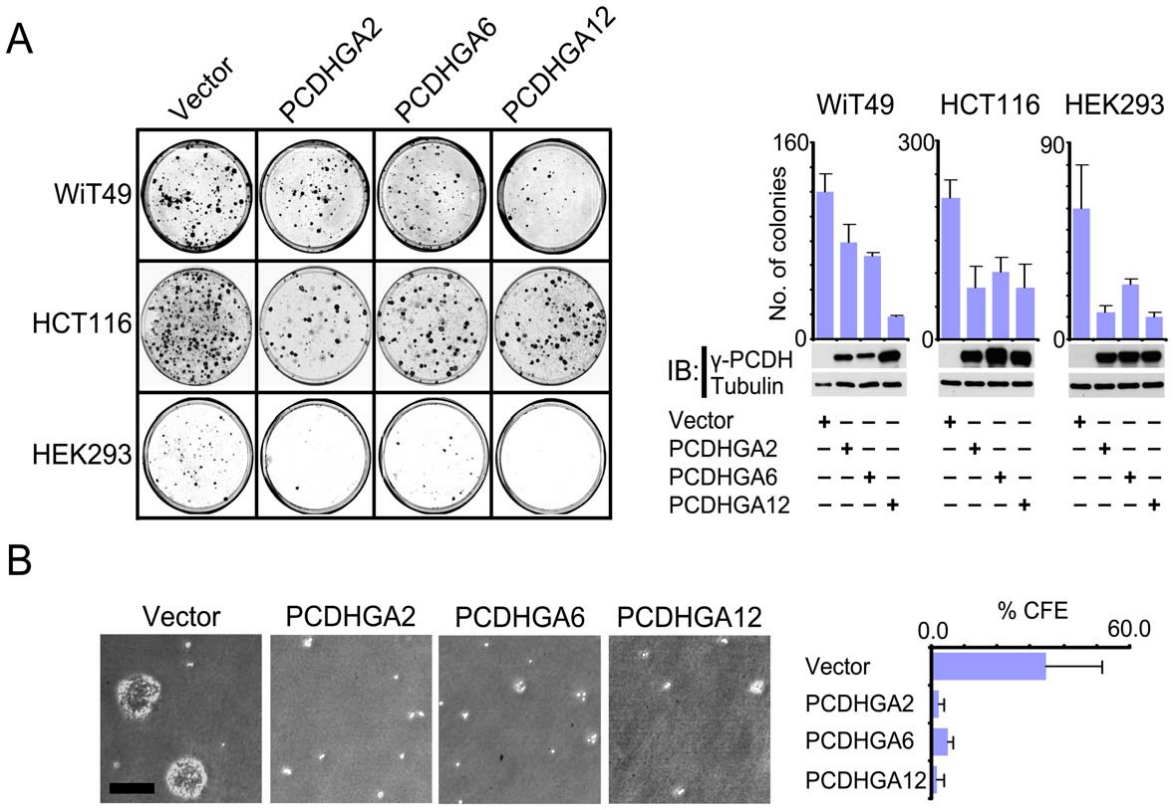
Growth inhibition by PCDHs. (A) Suppression of WiT49, HCT116, and HEK293 cell colony formation following ectopic expression of *PCDH* cDNAs. After selection and staining, plates were photographed and colony counts determined for each transfection. Representative plates (left) and mean colony counts (right) are shown, Verification of PCDH protein expression after transfection, together with tubulin as a loading control is shown below the histograms. (B) Inhibition of anchorage-independent growth of HCT116 cells by PCDHs, cells were plated in triplicate and colonies formed after 10–14 days were photographed and counted within 10 random fields. Representative fields are shown (left) together with colony forming efficiency (CFE), expressed as percentage of colonies >50 µm diameter (right). Cell-based assays were repeated at least twice, and representative data are shown.

As our epigenetic and functional analyses allude to a tumour suppressor function for PCDHs, we evaluated the effect of ectopic PCDH expression on tumour-related phenotype using cell-culture based assays commonly used for assessing tumour suppressor gene function, that is inhibition of colony formation and growth in soft agar [15]. Transfection of WiT49 cells with *PCDHGA2*, *PCDHGA6* or *PCDHGA12* resulted in moderate PCDH protein expression leading to approximately 30–85% suppression of colony formation (*P*<0.05 to *P*<0.005, *t*-test). Similarly transfection of HCT116 cells resulted in approximately 70% suppression of colony formation (*P*<0.01), and transfection of HEK293 cells led to 60–85% suppression (*P*<0.005) (Figure 6A). Suppression of colony formation was not a non-specific side-effect of gene over-expression, as transfection of HEK293 cells with *CTNNB1* cDNA encoding a degradation-resistant mutant of β-catenin failed to suppress colony formation (Figure S7).

We also assessed the effect of PCDH expression on anchorage independent growth in soft agar using HCT116 cells (Figure 6B). Colony forming efficiency was markedly reduced by PCDHGA2, PCDHGA6 and PCDHGA12 relative to cells transfected with expression vector only (approximately 85%–95% suppression relative to the vector control).

Collectively, therefore, our experiments demonstrate that PCDHs regulate critical transduction and transcription pathways and have growth regulatory properties consistent with tumour suppressor activity.

## Discussion

By conducting genome-wide promoter methylation analysis, we have identified a large cluster of paralogous *PCDH* genes on chromosome 5q31 which undergo hypermethylation in Wilms' tumours. Transcriptional silencing of *PCDH*s was prevalent in WTs, and *PCDH* hypermethylation constitutes the most frequent epigenetic silencing event in WT. This putative WT suppressor domain is the first report of LRES in childhood tumours. Our data also suggests roles for PCDHs in normal nephrogenesis, including modulation of key regulatory pathways such as canonical Wnt signalling.

LRES regions have been identified in several adult cancers including, breast [13], colon [11],[12], head, neck and lung [14],[28],[29]. A recent genome-wide analysis of methylation in breast cancers showed that multiple agglomerative epigenetic aberrations occur, including regions undergoing hypermethylation and hypomethylation. Interestingly, the *PCDH* locus on chromosome 5q31 was one of several hypermethylated domains shown in breast cancer, together with others such as *HOXD@* on chromosome 2 and *HIST1* on chromosome 6 [30]. Such regions were also identified in our analysis (Table S1). Thus, in addition to gene-specific epigenetic lesions, our study shows that some, but not all, LRES domains are conserved between embryonal and adult cancers. Also individual genes within an LRES region can show tumour-type specific changes, illustrated by *PCDHGC3* which is frequently hypermethylated in breast cancer but which escapes methylation in WTs. The contribution of LRES to tumour pathology is not well characterised, but transcriptional suppression of multiple genes across a chromosomal region can be considered to be functionally analogous to cytogenetic loss.

Silencing of individual genes within the LRES domains on chromosome 2q and 3p in colorectal cancer appears to be dependent on a domain-wide non-permissive chromatin configuration, rather than the methylation status, as unmethylated genes within these domains and up to 1000 kb away are also suppressed [11],[12]. By contrast, expression of non-*PCDH* genes at 5q31 (*TAF7* and *SLC25A2*) was strongly linked to methylation status in WTs, and the transcriptional status of unmethylated genes flanking the LRES was unchanged in tumours. A comparatively lesser degree of transcriptional suppression is observed for the unmethylated *PCDHGC3* and *PCDHGA6* genes in WiT49 cells. This suppression occurs despite any significant changes in active/repressive histone marks and suggests that *PCDHGA6* and *PCDHGC3* are repressed by a non-epigenetic effect such as altered feedback regulation resulting from lowered levels of γ-PCDH intracellular domain fragments. Similar to the Notch signalling paradigm, regulated presenilin dependent-processing of the γ-PCDHs generates C-terminal fragments which can localize to the nucleus and autoregulate the γ-*PCDH*s [20],[21].

In contrast to breast cancer [30], our ChIP data shows a strong link between DNA methylation and H3K9me2 at silenced *PCDH* genes, as reported for the LRES on chromosome 2q14.2 and 3p22 [11],[12]. Indeed a correlation between degree of silencing and H3K9me2 enrichment was apparent, whereas H3K4me2 and H3Ac marking is evident in all active genes and lost in silenced genes. This suggests that H3K9me2 plays a role in establishing and maintaining the silenced state, as previously demonstrated for *CDKN2A* [31], and that histone 3 acetylation and H3K4me2 marks are removed prior to increases in H3K9me2 and DNA methylation. It has been postulated that a significant proportion of hypermethylated loci in cancer do not arise by adaptive selection but rather are the result of an ‘instructive’ mechanism, via *cis*-targeting of the *trans*-acting Polycomb group protein-complexes [32], and that these loci are pre-marked in normal (unmethylated) tissues by histone H3 trimethyl - lysine27 (H3K27me3). The instructive mechanism may explain the non-random *de novo* methylation of some genes during tumorigenesis [33]. However, in the case of the 5q31 LRES, a genomic study of human embryonic stem cells failed to identify pre-marking of the hypermethylated *PCDHs* by Polycomb group proteins or H3K27me3 [34]. Additionally, the instructive mechanism predicts methylation would be present in pre-cancerous lesions (e.g. colorectal adenomas [32]) and we have shown this is not the case for *PCDHs*, which are unmethylated in nephrogenic rests, pre-cancerous lesions for WT. Therefore the *PCDH*s do not appear to be pre-marked for *de novo* methylation in WT, indicating that this molecular lesion is selected for during tumorigenesis. This is also supported by tumour-type specific variations in hypermethylation such as observed for *PCDHGC3*, as discussed above.

Hypermethylation of *PCDH*s was not detectable in nephrogenic rests, consistent with a previous assessment of *RASSF1*, *DNAJC15*/*MCJ* and *TNFRSF25* gene hypermethylation [35]. This is in contrast to gene-specific hypomethylation of the *GLIPR1* gene observed in WTs, where nephrogenic rests display intermediate methylation levels relative to fetal kidney and WTs [9]. Therefore although the *GLIPR1* hypomethylation observed in WTs might reflect an expansion of oncofetal cells lacking *GLIPR1* methylation, hypermethylation of *PCDHs* and other tumour suppressor genes appears to represent a later, tumour-specific lesion. Expression of PCDHs in blastemal cells, together with our methylation analysis of nephrogenic rests, also negates the possibility that Wilms' tumour *PCDH* hypermethylation can be attributed to clonal expansion of progenitors with cell-type specific methylation.

Genetic lesions in WT known to be late events include chromosome 16q loss of heterozygosity [36] and *CTNNB1* mutations [37]; interestingly, the CTCF gene locates to 16q, is mutated in some WTs [38] and the encoded epiregulatory protein has multiple binding sites across the *PCDH* locus [39], suggesting that aberrant CTCF function may be involved in LRES.

Expression of the *Pcdhs* peaks in the last week of nephrogenesis; thereafter, expression decreases, in contrast to *Cdh1*, which encodes the archetypal epithelial adhesion protein, E-cadherin. Epithelial differentiation of rat metanephric mesenchyme cells in organ culture was also accompanied by decreasing levels of *Pcdhg@* expression. A recent microarray analysis of gene expression with laser captured kidney components showed expression of *Pcdhb15* and *Pcdhga12* expression attenuating between the cap mesenchyme and renal vesicle [40]. Together with our expression analyses, this suggests that the *Pcdh* expression peak in murine nephrogenesis likely reflects the expansion of nephrogenic progenitors as kidney development nears completion [41]. Our *PCDH* expression analyses in human fetal kidney, during murine nephrogenesis and in rat metanehpric mesenchyme suggest that PCDHs may have hitherto uncharacterised roles in renal development. Although *Pcdhg@* mutant mice, which undergo neurodegeneration and neonatal death in less than 12 hours, did not show a gross kidney phenotype, kidney defects were not explored in detail [42] (Wang & Sanes, personal communication). The early postnatal lethality observed with homozygous *Pcdhg@* mutant mice would also preclude full assessment of effects on nephrogenesis, as murine kidney development continues in the first week following birth. Although we were unable to assess renal defects in *Pcdhg@* null mice, we did examine postnatal kidney from heterozygous *Pcdhg@* mutant mice (see Text S1), as heterozygous mutations of developmental genes such as *Wt1* have been shown to result in end-stage renal disease [43]. Histological examination of kidneys from 3 month old heterozygotes showed no evidence of overt kidney malformations (Figure S8). However, it will clearly be of great interest to analyse a larger heterozygous cohort together with embryonic kidney from homozygous *Pcdhg@* mutants in future studies.

The canonical Wnt signalling pathway is a prerequisite for initiating and maintaining mesenchymal to epithelial transitions during kidney development, and it is also known that mesenchyme with high β-catenin activity fails to form epithelial structures [44]. Thus attenuation of Wnt signalling is necessary during nephrogenesis. Importantly, our functional analysis shows that γ-PCDHs repress β-catenin/TCF mediated transcription, with lowered PCDH leading to elevated β-catenin protein, high β-catenin/TCF reporter activity and induced expression of Wnt target genes. Conversely, ectopic expression of *PCDHs* was able to suppress β-catenin/TCF reporter activity in heterologous cell systems. In contrast to Wnt target genes, *WT1* expression levels were reduced by PCDH knockdown, demonstrating that Wnt target gene induction is not reflecting a generalised increase in transcription and that other regulatory networks are also influenced by cellular PCDH levels. The significance of these results is underlined by our findings of epigenetic silencing of *PCDH*s in WT, as this would be predicted to lead to elevated β-catenin/TCF activity. In this regard, it is notable that enhanced β-catenin signalling in WTs is observed more frequently than *CTNNB1* and *WTX* mutations in WTs [45],[46] and that *CTNNB1* mutation is, like *PCDH* silencing, a late event in Wilms' tumorigenesis. Although our analysis of PCDHs on Wnt signalling can only approximate the permutational silencing in WTs, our results prompt the hypothesis that the canonical Wnt pathway is modulated by PCDHs, and that in normal nephrogenesis, elevated PCDHs serve to downregulate β-catenin activity, thereby permitting completion of epithelial differentiation. Epigenetic silencing of *PCDH*s might contribute to deregulation of Wnt signalling, and a failure of mesenchymal to epithelial transition resulting in persistence of a progenitor cell pool and consequent Wilms' tumorigenesis. PCDHs may also have a role in the aetiology of other cancers, such as breast cancer, where *PCDH* hypermethylation is prevalent [30] and activation of Wnt/β-catenin signalling occurs, despite mutations of Wnt pathway components being rare [47].

Although the mechanisms by which PCDHs influence pathways such as Wnt signalling require delineation, we note that, as well as encoding a nuclear moiety capable of regulating gene expression directly [20],[21], α- and γ-PCDHs have recently been reported to negatively regulate proline-rich tyrosine kinase 2 (PYK2) [48] which was previously shown to phosphorylate β-catenin [49]. As phospho-regulation of β-catenin can promote interactions with transcriptional co-activators [50], we speculate that elevated PYK2 activity may arise as a consequence of *PCDH* silencing and thereby lead to a shift of the β-catenin adhesion/signalling balance. This and other downstream consequences of *PCDH* silencing warrant intensive future study.

## Materials and Methods

### Ethical statement

All human tissues were acquired with appropriate local research ethics committee approval and all animal procedures were conducted in accordance with regulations (UK Home Office) and international standards on animal welfare.

### Patient samples

All tissues were obtained as snap frozen samples from the Bristol Children's Hospital, the Royal Marsden Hospital and the University of Heidelberg Children's Hospital. Human fetal tissue samples were from 19 to 31 weeks of gestation. Details of clinical samples are given in Table S3.

### Cell culture, transfections, and reporter assays

WiT49 [18], HCT116 [9] and HEK293 cell-lines (adenovirus transformed human embryonic kidney cells [51]) were cultured using standard methods in Dulbecco's modified Eagle's medium (DMEM) supplemented with 10% fetal calf serum, 2 mmol/l L-glutamine, 0.1 mg/ml penicillin/streptomycin, at 37°C under 5% CO_2_. L/Wnt3a fibroblast cell lines (ATCC, Manassas, VA) were grown in DMEM supplemented with 10% fetal calf serum, and Wnt3a conditioned medium was prepared according to the protocol provided by ATCC (http://www.atcc.org).

For knockdown analyses, cells were transfected with DharmaFECT DUO (Dharmacon, Inc.) with 50 nM total of ON-TARGETplus short interfering RNA (siRNA) duplexes. For β-catenin/TCF reporter activity assays, 10^5^ cells/well were seeded in 24-well plates and 100 ng of Super 8xTOPFLASH or Super 8xFOPFLASH reporter plasmids were co-transfected with siRNAs and 100 pg of pRL-SV40 to normalise for transfection efficiency. Super 8xFOPFLASH is a negative control for Super 8xTOPFLASH containing mutated TCF binding sites [25]. Luciferase samples were assayed after 48 hours using Dual-luciferase reporter kit (Promega) and a Modulus Luminometer (Turner Biosystems). Experiments were performed at least twice in triplicate.

For rat mesenchymal organ culture, dissected fresh metanephric mesenchyme from E13.5 rat embryos was cultured on collagen coated transwell filters (Corning) in DMEM/F12 media containing 20 mg/ml penicillin/streptomycin, 10 µg/ml insulin, 10 µg/ml transferrin, 10 ng/ml selenium (ITS), 40 ng/ml dexamethasone, 100 ng/ml prostaglandin, 4 ng/ml tri-iodo-l-thyronine, 10 ng/ml holo-transferrin. Epithelial differentiation was induced over 5 days with 50 ng/ml leukemia inhibitory factor (Lif, Chemicon), 20 ng/ml transforming growth factor α (Tgfα, R&D Systems) and 50 ng/ml fibroblast growth factor (Fgf2, R&D Systems) [19], after which RNA was extracted with Tri reagent (Sigma).

For PCDH over-expression studies, cDNAs were obtained by RT-PCR from fetal kidney RNA and cloned into pCDNA3.1/Zeo (Invitrogen). For β-catenin/TCF reporter assays, 10^5^ cells/well were seeded in 24-well plates and transfected with 400 ng of expression constructs together with 100 ng Super 8xTOPFLASH or Super 8xFOPFLASH using Fugene 6 (Roche), and assayed for luciferase as described above. For colony formation assays, 2.5×10^5^ HCT116 and WiT49 cells were transfected with 1 ug of expression plasmids in 6-well plates and plated in triplicate in 10 cm dishes 48 hours after transfection. Selection was performed with 200 µg/ml (HCT116) or 100 µg/ml (WiT49) of zeocin (Invitrogen) for 2 weeks after which colonies were methylene blue stained and counted using ImageJ software (http://rsbweb.nih.gov/ij/). Colony formation assays were repeated at least twice.

Growth in soft agar was assessed essentially as previously described [15]. Briefly, 2.5×10^4^ transfected HCT116 cells were suspended in DMEM medium containing 10% fetal calf serum, 0.35% agar, and 200 µg/ml zeocin. The suspension was then layered on 6 cm plates containing DMEM medium containing 10% fetal calf serum, 0.7% agar, and 200 µg/ml zeocin. Plating was carried out in triplicate and repeated at least twice. Cells were fed every 4–5 days, and after 10–14 days growth, colonies of greater than approximately 50 µm within 10 microscopic fields were counted under a phase contrast microscope. Colony forming efficiency is presented as percentage of colonies larger than 50 µm within total cells.

### Methylated–DNA immunoprecipitation (MeDIP) and chromatin immunoprecipitation (ChIP)

High molecular weight genomic DNAs were extracted from tissues using standard phenol-chloroform techniques and fragmented to a size range of 200–500 base pairs using a Diagenode Bioruptor. Four micrograms of sonicated genomic DNA and 20 µg anti-5-methyl cytidine monoclonal antibody (Eurogentec, Liège, Belgium) were incubated at 4°C overnight in immunoprecipitation buffer, and then for a further 2 hours with goat anti-mouse IgG magnetic beads (N.E. Biolabs). After purification, MeDIP DNA was blunt-ended with T4 DNA polymerase (N.E. Biolabs) and ligation-mediated PCR (LM-PCR) was carried out as described [52],[53]. DNAs were then sent to Nimblegen for labelling and hybridization to Nimblegen HG18 Refseq promoter arrays. Data was analysed using ChipMonk v1.2.1 tiling array analysis software (Dr Simon Andrews, Babraham Institute, Cambridge UK, http://www.bioinformatics.bbsrc.ac.uk/projects/chipmonk). To identify hypermethylated CGIs, a log2ratio cut-off of 1.5 and a window of 500 bp was used to carry out the replicate *t*-test (*P*<0.05) on probes within 200 bp of predicted CpG islands (http://genome.ucsc.edu). Additional statistical analysis was carried out using R (http://www.r-project.org/). The array data reported in this paper have been deposited in the Gene Expression Omnibus (GEO) database, (http://www.ncbi.nlm.nih.gov/geo) under accession number GSE15027.

WiT49 chromatin marks were assessed using a ChIP Kit (Upstate Biotechnology) with antibodies for histone 3 dimethyl lysine 4 (H3K4me2, Upstate Technology), histone 3 dimethyl lysine 9 (H3K9me2, Abcam), and histone 3 acetyl lysine (H3Ac, Upstate). Quantification using real-time PCR was carried out using the Stratagene MX3005P QPCR System (La Jolla, CA) along with the PlatinumSYBR green qPCR SuperMix-UDG (Invitrogen). Reactions volumes of 20 µl contained 10 µl of Platinum SYBRgreen qPCR SuperMix-UDG (Invitrogen, Paisley, UK), 50 nM ROX reference dye, 0.2 µM forward primer, 0.2 µM reverse primer, and 1.5 µl of ChIP DNA template. Primer sequences are available in Table S4.

### Methylation and expression analysis

Up to 1 µg DNA was sodium bisulfite converted using the EZ DNA Methylation-Gold Kit (Zymo Research, CA). Amplicons for combined bisulfite restriction analysis (COBRA) and bisulfite sequencing were made using the Hot Start Red Taq PCR system (Sigma). Primers and restriction enzymes are available in Table S4. For bisulfite sequencing, PCR products were cloned into the pGEM-T-easy cloning vector (Promega), and fluorescently sequenced using standard M13 primer sequences.

For comparative quantitative Real-Time RT-PCR, 1 µg of DNAse-treated (TURBO DNA-free, Ambion Inc, TX) total RNA was reverse-transcribed with oligo(dT)_20_ at 50°C for 1 hr using the ThermoScript RT-PCR System (Invitrogen). Real-time PCR was performed using the Stratagene MX3005P QPCR System (La Jolla, CA) along with the PlatinumSYBR green qPCR SuperMix-UDG (Invitrogen). Reactions volumes of 20 µl contained 10 µl of Platinum SYBRgreen qPCR SuperMix-UDG (Invitrogen, Paisley, UK), 50 nM ROX reference dye, 0.2 µM forward primer, 0.2 µM reverse primer, and 2.5 µl of 1∶10 diluted cDNA template. Primer sequences are available in Table S4. Thermal cycling consisted of an initial incubation step of 50°C for 2 minutes and a denaturation step of 95°C for 10 minutes. This was followed by 40 cycles of 95°C/15 seconds, 58°C/30 seconds, 72°C/30 seconds. Gene expression was quantified by comparative Ct method, normalizing values to the housekeeping gene TBP. All assays were performed in duplicate.

### Immunoblotting and immunohistochemistry

Protein extraction and immunoblotting were carried out essentially as previously described [2] with primary antibodies to β-catenin (Cell signalling), α-tubulin (Sigma) and the γ-PCDH constant region (Greg Phillips, Mount Sinai School of Medicine, USA). This pan γ-PCDH antibody is a characterised affinity-purified rabbit polyclonal against a GST-fusion protein containing the constant cytoplasmic domain encoded by *PCDHG@* [54],[55]. This region is highly conserved in human and mouse, with one amino acid difference in 124 amino-acids. Briefly, tissues were lysed in 300 µl of sample buffer (60 mM Tris pH 6.8, 10% glycerol, 2% SDS, 5% mercaptoethanol) and 10 µg protein was loaded per well and electrophoresed on a 10% SDS – polyacrylamide gel. After electrophoresis the proteins were transferred to Immobilon-P (Millipore) with a semidry transfer apparatus. The Immobilon-P was then transferred to 5% non-fat dry milk (Tesco) in PBS (milk block) and blocked for a minimum of 1 hour. Primary antibodies were incubated in milk block overnight at 4°C, followed by the secondary antibody at room temperature for an hour. Protein bands were visualised with ECL Plus reagents (Amersham Pharmacia Biotech).

For immunohistochemistry, 5 µm sections of CBA x C57Bl/6 F2 mouse P0 neonatal kidney were fixed for 16 hrs in 4% paraformaldehyde. Antigen retrieval of deparaffinised sections was performed by microwaving in 0.8 M urea, pH 6.4, followed by indirect immunoperoxidase staining using the Elite ABC kit (Rabbit IgG; Vectastain) according to the manufacturer's instructions. Primary antibody dilutions were 1∶100 for WT1 (6FH2, Dako), and 1∶200 for pan γ-PCDH. Sections were counterstained with haematoxylin. Negative control sections omitted the primary antibody.

## Supporting Information

Figure S1 Analysis of aberrant methylation in Wilms' tumours using Nimblegen Refseq promoter HG18 tiling arrays. (A) The MeDIP-chip workflow used to analyse genome-wide methylation. Methylated DNA purified from normal and tumour DNAs was amplified using ligation-mediated PCR (LM-PCR), labelled and hybridised with promoter microarrays (B) Real-time RT-PCR of MeDIP-enriched tumour DNAs (upper). Enrichment was validated using primers specific to a non-CGI sequence within the *TBP* gene (grey bars), the constitutively methylated *H19* imprinting control region (black bars), and selective enrichment of the methylated *RASSF1* 5′-CGI in a methylated and unmethylated tumour (open bars). MeDIP DNA was quantified relative to input DNA. The lower panel shows COBRA confirmation of *H19* imprinting control region and the *RASSF1* 5′-CGI methylation status. Arrowheads show methylated (M) and unmethylated (UM) DNA fragments; presence or absence of restriction enzyme is indicated (+/−). (C) The tumour (T) versus normal fetal kidney (N) signal ratio (y-axis) from 17,777 promoter-associated probes are plotted according to their physical location on chromosome 5 (x-axis) for 5 WTs. Hypermethylation at 5q31 is indicated by the vertical arrow.
(4.67 MB TIF)

Figure S2 Methylation analysis of genes neighbouring the chromosome 5q31 *PCDH* cluster. Arrowheads show methylated (M) and unmethylated (UM) DNA fragments; presence or absence of restriction enzyme is indicated (+/−).M+, in vitro methylated DNA (A) Distal neighbours of the clustered PCDHs in normal and tumour tissues. COBRA analysis of *CD14, TMCO6, NDUFA2* and *WDR55* 5′-CGIs (located -153, -147, -139, and -119 kbp upstream of the *PCDH* clusters, respectively). FK, 22-week fetal kidney; WTs, five pooled WT DNAs. (B) 5′-CGI methylation analysis of the non-clustered *PCDH1* gene (located 366 kbp downstream of the *PCDH* clusters) was carried out on eleven WTs using COBRA. 22-week foetal kidney, FK; FK2, 16-week foetal kidney.
(1.85 MB TIF)

Figure S3 Methylation analysis of *PCDHB6* in WT precursor lesions. (A) COBRA analysis of *PCDHB6* in DNA extracted from fetal kidney (FK), WTs, and associated perilobar nephrogenic rests (NR). T, Wilms' tumours. Arrowheads show methylated (M) and unmethylated (UM) DNA fragments; presence or absence of restriction enzyme is indicated (+/−). M+, in vitro methylated DNA. (B) Bisulfite sequencing analysis. Black circles represent methylated CpGs and white circles represent unmethylated CpGs.
(1.22 MB TIF)

Figure S4 *PCDH* hypermethylation in stromal-predominant Wilms' tumours. COBRA was carried out for *PCDHGA3, PCDHGB4*, and *HDAC3*. Arrowheads show methylated (M) and unmethylated (UM) DNA fragments; presence or absence of restriction enzyme is indicated (+/−). M+, in vitro methylated DNA.
(2.02 MB TIF)

Figure S5 Pharmacological demethylation of WiT49 cells. Quantitative real-time RT-PCR of 5q31 transcripts, mock-treated (-) or 5-azacytidine treated (+) cells. Grey bars indicate genes associated with hypermethylated CGIs, white bars represent genes with CpG islands with no detectable methylation and black bars are used for *SLC25A2. HPRT* is an X-chromosome housekeeping control gene. *DIAPH1* and *HDAC3* are located on chromosome 5q31 outside the LRES. Expression data for 3 *PCDHB@* genes and 4 *PCDHG@* genes is shown relative to *TBP*, together with *SLC25A2* and *TAF7* genes, which are located within the LRES. Induction of the WT hypermethylated control genes *RASSF1* and *H19* is also shown.
(2.41 MB TIF)

Figure S6 Hypermethylation of *PCDHGA2, PCDHGA6, PCDHGA12, PCDHB6, PCDHGC3* and *PCDHGA7* in HCT116 cells demonstrated using COBRA analysis. Arrowheads show methylated (M) and unmethylated (UM) DNA fragments; presence or absence of restriction enzyme is indicated (+/−).
(1.25 MB TIF)

Figure S7 Suppression of colony formation is not dependent on non-specific toxicity of transfected genes. Mutant β-catenin (Y33, tyrosine at amino-acid 33) expression does not suppress colony formation in HEK293 cells. HEK293 cells were transfected with *CTNNB1* cDNA cloned in the same expression vector (pcDNA3.1/Zeo) as *PCDH* constructs. After selection and staining, plates were photographed and colony counts determined for each transfection. Representative plates (above) and mean colony counts (below) are shown. Verification of β-catenin protein expression after transfection is shown by immunoblotting below the histograms, together with tubulin to control loading.
(1.35 MB TIF)

Figure S8 Kidneys of heterozygous *Pcdhg@* mutant mice show no malformations (see Text S1). Histology of three-month old wild-type (wt, n = 2) and heterozygous *Pcdhg@* mutant kidneys (het, n = 3) was examined on cryosections. Staining of adjacent sections with cresyl-violet (left column) and nuclear fast red (middle and right columns) was used to highlight the cytoarchitecture of the specimens. The overall morphology of the heterozygous kidneys appeared normal and showed no malformations. Scale bars  = 500 µm. At higher magnifications, findings were comparable in three-month old wild-type and heterozygous littermates and displayed normal cytoarchitecture in aged heterozygous mice (boxed areas are shown in the right column, scale bars  = 100 µm).
(1.02 MB TIF)

Table S1 Wilms' tumour hypermethylated genes identified by MeDIP-chip. Summary table of hypermethylated genes identified by MeDIP-chip in five Wilms' tumours (*P*<0.05). Ensembl gene ID, gene symbol, and co-ordinates by chromosome and gene start (HG18 genome build) are given. The frequency of hypermethylation is given in the final column. Blanks in the Gene name column represent Refseq unannotated genes.
(0.23 MB XLS)

Table S2 COBRA methylation summary. Methylation data from normal and tumour samples ascertained by combined bisulphite restriction analysis (COBRA). U, unmethylated; M, predominantly methylated; m, partially methylated. L and R, tumours of the left and right kidneys, respectively.
(0.04 MB XLS)

Table S3 Clinical details of nephrogenic rests and Wilms' tumours. Age at diagnosis: m, age in months. Histology: FH, favorable histology; UH, unfavorable histology; TR, triphasic; B, blastemal predominant; E; epithelial predominant; S, stromal predominant; A, anaplastic; T, teratoid; R, regressive. Outcome: A, alive; R, relapsed; D, died. *WT1* mutation: Y, yes; N, no. *WT1* mutation details: G/L, germline. Blank entry, not done.
(0.02 MB XLS)

Table S4 Oligonucleotide primers used in this study.
(0.04 MB XLS)

Text S1 Supporting information methods.
(0.03 MB DOC)

## References

[1] Rivera MN, Haber DA (2005). Wilms' tumour: connecting tumorigenesis and organ development in the kidney.. Nat Rev Cancer.

[2] Dallosso AR, Hancock AL, Brown KW, Williams AC, Jackson S (2003). Genomic Imprinting at the WT1 gene involves a novel coding transcript (AWT1) that shows deregulation in Wilms' tumours.. Hum Mol Genet.

[3] Malik K, Salpekar A, Hancock A, Moorwood K, Jackson S (2000). Identification of differential methylation of the WT1 antisense regulatory region and relaxation of imprinting in Wilms' tumor.. Cancer Res.

[4] Ogawa O, Eccles MR, Szeto J, McNoe LA, Yun K (1993). Relaxation of Insulin-Like Growth Factor-II Gene Imprinting Implicated in Wilms' Tumour.. Nature.

[5] Brown KW, Power F, Moore B, Charles AK, Malik KT (2008). Frequency and timing of loss of imprinting at 11p13 and 11p15 in Wilms' tumor development.. Mol Cancer Res.

[6] Zhang L, Anglesio MS, O'Sullivan M, Zhang F, Yang G (2007). The E3 ligase HACE1 is a critical chromosome 6q21 tumor suppressor involved in multiple cancers.. Nat Med.

[7] Wagner KJ, Cooper WN, Grundy RG, Caldwell G, Jones C (2002). Frequent RASSF1A tumour suppressor gene promoter methylation in Wilms' tumour and colorectal cancer.. Oncogene.

[8] Morris MR, Hesson LB, Wagner KJ, Morgan NV, Astuti D (2003). Multigene methylation analysis of Wilms' tumour and adult renal cell carcinoma.. Oncogene.

[9] Chilukamarri L, Hancock AL, Malik S, Zabkiewicz J, Baker JA (2007). Hypomethylation and aberrant expression of the glioma pathogenesis-related 1 gene in Wilms tumors.. Neoplasia.

[10] Morishita H, Yagi T (2007). Protocadherin family: diversity, structure, and function.. Curr Opin Cell Biol.

[11] Frigola J, Song J, Stirzaker C, Hinshelwood RA, Peinado MA (2006). Epigenetic remodeling in colorectal cancer results in coordinate gene suppression across an entire chromosome band.. Nat Genet.

[12] Hitchins MP, Lin VA, Buckle A, Cheong K, Halani N (2007). Epigenetic inactivation of a cluster of genes flanking MLH1 in microsatellite-unstable colorectal cancer.. Cancer Res.

[13] Novak P, Jensen T, Oshiro MM, Wozniak RJ, Nouzova M (2006). Epigenetic inactivation of the HOXA gene cluster in breast cancer.. Cancer Res.

[14] Rauch T, Wang Z, Zhang X, Zhong X, Wu X (2007). Homeobox gene methylation in lung cancer studied by genome-wide analysis with a microarray-based methylated CpG island recovery assay.. Proc Natl Acad Sci U S A.

[15] Ying J, Li H, Seng TJ, Langford C, Srivastava G (2006). Functional epigenetics identifies a protocadherin PCDH10 as a candidate tumor suppressor for nasopharyngeal, esophageal and multiple other carcinomas with frequent methylation.. Oncogene.

[16] Yu J, Cheng YY, Tao Q, Cheung KF, Lam CN (2009). Methylation of protocadherin 10, a novel tumor suppressor, is associated with poor prognosis in patients with gastric cancer.. Gastroenterology.

[17] Yu JS, Koujak S, Nagase S, Li CM, Su T (2008). PCDH8, the human homolog of PAPC, is a candidate tumor suppressor of breast cancer.. Oncogene.

[18] Alami J, Williams BR, Yeger H (2003). Derivation and characterization of a Wilms' tumour cell line, WiT 49.. Int J Cancer.

[19] Schmidt-Ott KM, Masckauchan TNH, Chen X, Hirsh BJ, Sarkar A (2007). beta-catenin/TCF/Lef controls a differentiation-associated transcriptional program in renal epithelial progenitors.. Development.

[20] Haas IG, Frank M, Veron N, Kemler R (2005). Presenilin-dependent processing and nuclear function of gamma-protocadherins.. J Biol Chem.

[21] Hambsch B, Grinevich V, Seeburg PH, Schwarz MK (2005). Gamma-Protocadherins, presenilin-mediated release of C-terminal fragment promotes locus expression.. J Biol Chem.

[22] Yang XZ, Chen MW, Terry S, Vacherot F, Chopin DK (2005). A human- and male-specific protocadherin that acts through the Wnt signaling pathway to induce neuroendocrine transdifferentiation of prostate cancer cells.. Cancer Res.

[23] Schmidt-Ott KM, Barasch J (2008). WNT/beta-catenin signaling in nephron progenitors and their epithelial progeny.. Kidney Int.

[24] Giles RH, van Es JH, Clevers H (2003). Caught up in a Wnt storm: Wnt signaling in cancer.. Biochim Biophys Acta.

[25] Veeman MT, Slusarski DC, Kaykas A, Louie SH, Moon RT (2003). Zebrafish prickle, a modulator of noncanonical Wnt/Fz signaling, regulates gastrulation movements.. Curr Biol.

[26] Brattain MG, Fine WD, Khaled FM, Thompson J, Brattain DE (1981). Heterogeneity of malignant cells from a human colonic carcinoma.. Cancer Res.

[27] Ilyas M, Tomlinson IP, Rowan A, Pignatelli M, Bodmer WF (1997). Beta-catenin mutations in cell lines established from human colorectal cancers.. Proc Natl Acad Sci U S A.

[28] Smith LT, Lin M, Brena RM, Lang JC, Schuller DE (2006). Epigenetic regulation of the tumor suppressor gene TCF21 on 6q23-q24 in lung and head and neck cancer.. Proc Natl Acad Sci U S A.

[29] Liu Z, Zhao J, Chen XF, Li W, Liu R (2008). CpG island methylator phenotype involving tumor suppressor genes located on chromosome 3p in non-small cell lung cancer.. Lung Cancer.

[30] Novak P, Jensen T, Oshiro MM, Watts GS, Kim CJ (2008). Agglomerative epigenetic aberrations are a common event in human breast cancer.. Cancer Res.

[31] Bachman KE, Park BH, Rhee I, Rajagopalan H, Herman JG (2003). Histone modifications and silencing prior to DNA methylation of a tumor suppressor gene.. Cancer Cell.

[32] Keshet I, Schlesinger Y, Farkash S, Rand E, Hecht M (2006). Evidence for an instructive mechanism of de novo methylation in cancer cells.. Nat Genet.

[33] Schlesinger Y, Straussman R, Keshet I, Farkash S, Hecht M (2007). Polycomb-mediated methylation on Lys27 of histone H3 pre-marks genes for de novo methylation in cancer.. Nat Genet.

[34] Lee, TI (2006). Control of developmental regulators by Polycomb in human embryonic stem cells,. Cell.

[35] Ehrlich M, Jiang G, Fiala E, Dome JS, Yu MC (2002). Hypomethylation and hypermethylation of DNA in Wilms tumors.. Oncogene.

[36] Charles AK, Brown KW, Berry PJ (1998). Microdissecting the genetic events in nephrogenic rests and Wilms' tumor development.. Am J Path.

[37] Fukuzawa R, Heathcott RW, More HE, Reeve AE (2007). Sequential WT1 and CTNNB1 mutations and alterations of beta-catenin localisation in intralobar nephrogenic rests and associated Wilms tumours: two case studies.. J Clin Pathol.

[38] Filippova GN, Qi CF, Ulmer JE, Moore JM, Ward MD (2002). Tumor-associated zinc finger mutations in the CTCF transcription factor selectively alter its DNA-binding specificity.. Cancer Res.

[39] Kim TH, Abdullaev ZK, Smith AD, Ching KA, Loukinov DI (2007). Analysis of the vertebrate insulator protein CTCF-binding sites in the human genome.. Cell.

[40] Brunskill EW, Aronow BJ, Georgas K, Rumballe B, Valerius MT (2008). Atlas of gene expression in the developing kidney at microanatomic resolution.. Dev Cell.

[41] Kobayashi A, Valerius MT, Mugford JW, Carroll TJ, Self M (2008). Six2 defines and regulates a multipotent self-renewing nephron progenitor population throughout mammalian kidney development.. Cell Stem Cell.

[42] Wang XZ, Weiner JA, Levi S, Craig AM, Bradley A (2002). Gamma protocadherins are required for survival of spinal interneurons.. Neuron.

[43] Menke AL, Ijpenberg A, Fleming S, Ross A, Medine CN (2003). The wt1-heterozygous mouse; a model to study the development of glomerular sclerosis.. J Pathol.

[44] Park JS, Valerius MT, McMahon AP (2007). Wnt/beta-catenin signaling regulates nephron induction during mouse kidney development.. Development.

[45] Ruteshouser EC, Robinson SM, Huff V (2008). Wilms tumor genetics: mutations in WT1, WTX, and CTNNB1 account for only about one-third of tumors.. Genes Chromosomes Cancer.

[46] Koesters R, Ridder R, Kopp-Schneider A, Betts D, Adams V (1999). Mutational activation of the beta-catenin proto-oncogene is a common event in the development of Wilms' tumors.. Cancer Res.

[47] Howe LR, Brown AM (2004). Wnt signaling and breast cancer.. Cancer Biol Ther.

[48] Chen J, Lu Y, Meng S, Han MH, Lin C (2009). Alpha- and Gamma-Protocadherins negatively regulate PYK2.. J Biol Chem.

[49] van Buul JD, Anthony EC, Fernandez-Borja M, Burridge K, Hordijk PL (2005). Proline-rich tyrosine kinase 2 (Pyk2) mediates vascular endothelial-cadherin-based cell-cell adhesion by regulating beta-catenin tyrosine phosphorylation.. J Biol Chem.

[50] Daugherty RL, Gottardi CJ (2007). Phospho-regulation of Beta-catenin adhesion and signaling functions.. Physiology.

[51] Graham FL, Smiley J, Russell WC, Nairn R (1977). Characteristics of a human cell line transformed by DNA from human adenovirus type 5.. J Gen Virol.

[52] Weber M, Davies JJ, Wittig D, Oakeley EJ, Haase M (2005). Chromosome-wide and promoter-specific analyses identify sites of differential DNA methylation in normal and transformed human cells.. Nat Genet.

[53] Squazzo SL, O'Geen H, Komashko VM, Krig SR, Jin VX (2006). Suz12 binds to silenced regions of the genome in a cell-type-specific manner.. Genome Res.

[54] Phillips GR, Tanaka H, Frank M, Elste A, Fidler L (2003). Gamma-protocadherins are targeted to subsets of synapses and intracellular organelles in neurons.. J Neurosci.

[55] Frank M, Ebert M, Shan WS, Phillips GR, Arndt K (2005). Differential expression of individual gamma-protocadherins during mouse brain development.. Mol Cell Neurosci.

