# Correction: Frequent Long-Range Epigenetic Silencing of Protocadherin Gene Clusters on Chromosome 5q31 in Wilms' Tumor

**DOI:** 10.1371/annotation/012d5a44-8239-4057-8c3b-3dc159ea3a02

**Published:** 2009-12-04

**Authors:** Anthony R. Dallosso, Anne L. Hancock, Marianna Szemes, Kim Moorwood, Laxmi Chilukamarri, Hsin-Hao Tsai, Abby Sarkar, Jonathan Barasch, Raisa Vuononvirta, Chris Jones, Kathy Pritchard-Jones, Brigitte Royer-Pokora, Sean Bong Lee, Ceris Owen, Sally Malik, Yi Feng, Marcus Frank, Andrew Ward, Keith W. Brown, Karim Malik

There was an error in the author contributions. The author contributions should state that Keith W. Brown (KW Brown) and Karim Malik (K Malik) devised array analysis.

