# Supplementary figures and images for: Frequent Long-Range Epigenetic Silencing of Protocadherin Gene Clusters on Chromosome 5q31 in Wilms' Tumor
Source: PLoS Genet. 2009 Nov 26;5(11):e1000745. doi: 10.1371/journal.pgen.1000745 (PMC2776977; doi:10.1371/journal.pgen.1000745)

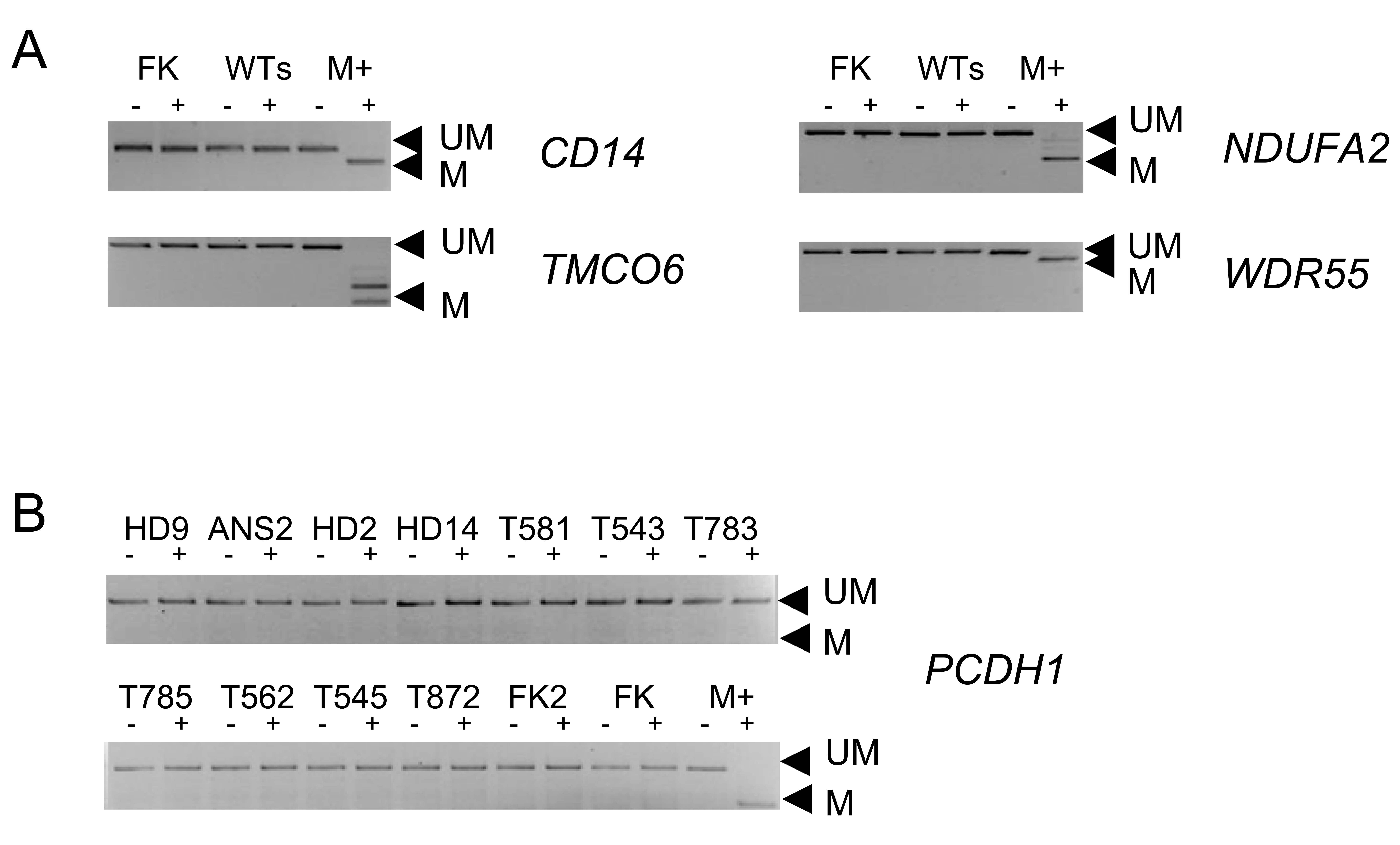

Supplement: Figure S2 — Methylation analysis of genes neighbouring the chromosome 5q31 PCDH cluster. Arrowheads show methylated (M) and unmethylated (UM) DNA fragments; presence or absence of restriction enzyme is indicated (+/−).M+, in vitro methylated DNA (A) Distal neighbours of the clustered PCDHs in normal and tumour tissues. COBRA analysis of CD14, TMCO6, NDUFA2 and WDR55 5′-CGIs (located -153, -147, -139, and -119 kbp upstream of the PCDH clusters, respectively). FK, 22-week fetal kidney; WTs, five pooled WT DNAs. (B) 5′-CGI methylation analysis of the non-clustered PCDH1 gene (located 366 kbp downstream of the PCDH clusters) was carried out on eleven WTs using COBRA. 22-week foetal kidney, FK; FK2, 16-week foetal kidney. (1.85 MB TIF) [file pgen.1000745.s002.tif]

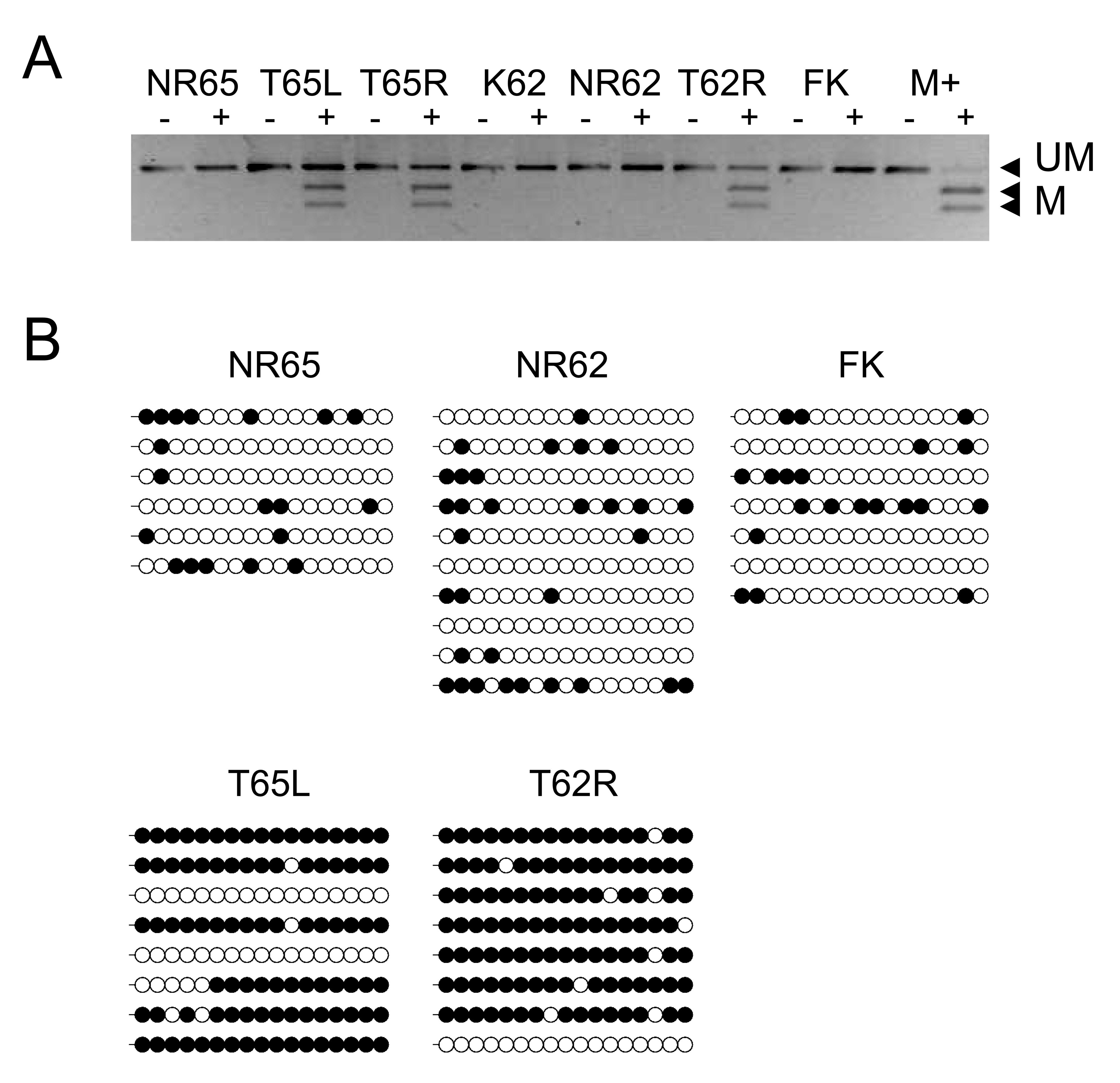

Supplement: Figure S3 — Methylation analysis of PCDHB6 in WT precursor lesions. (A) COBRA analysis of PCDHB6 in DNA extracted from fetal kidney (FK), WTs, and associated perilobar nephrogenic rests (NR). T, Wilms' tumours. Arrowheads show methylated (M) and unmethylated (UM) DNA fragments; presence or absence of restriction enzyme is indicated (+/−). M+, in vitro methylated DNA. (B) Bisulfite sequencing analysis. Black circles represent methylated CpGs and white circles represent unmethylated CpGs. (1.22 MB TIF) [file pgen.1000745.s003.tif]

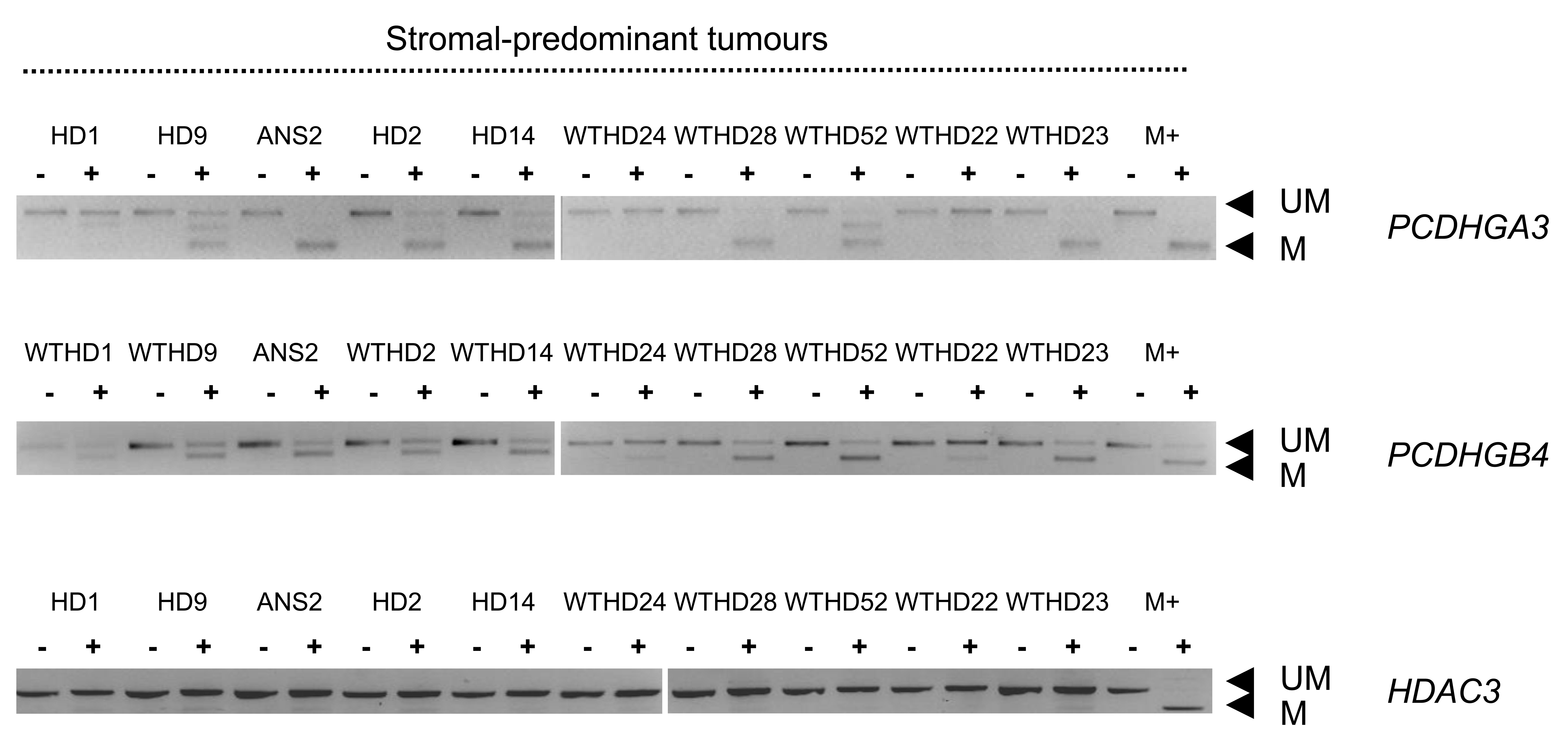

Supplement: Figure S4 — PCDH hypermethylation in stromal-predominant Wilms' tumours. COBRA was carried out for PCDHGA3, PCDHGB4, and HDAC3. Arrowheads show methylated (M) and unmethylated (UM) DNA fragments; presence or absence of restriction enzyme is indicated (+/−). M+, in vitro methylated DNA. (2.02 MB TIF) [file pgen.1000745.s004.tif]

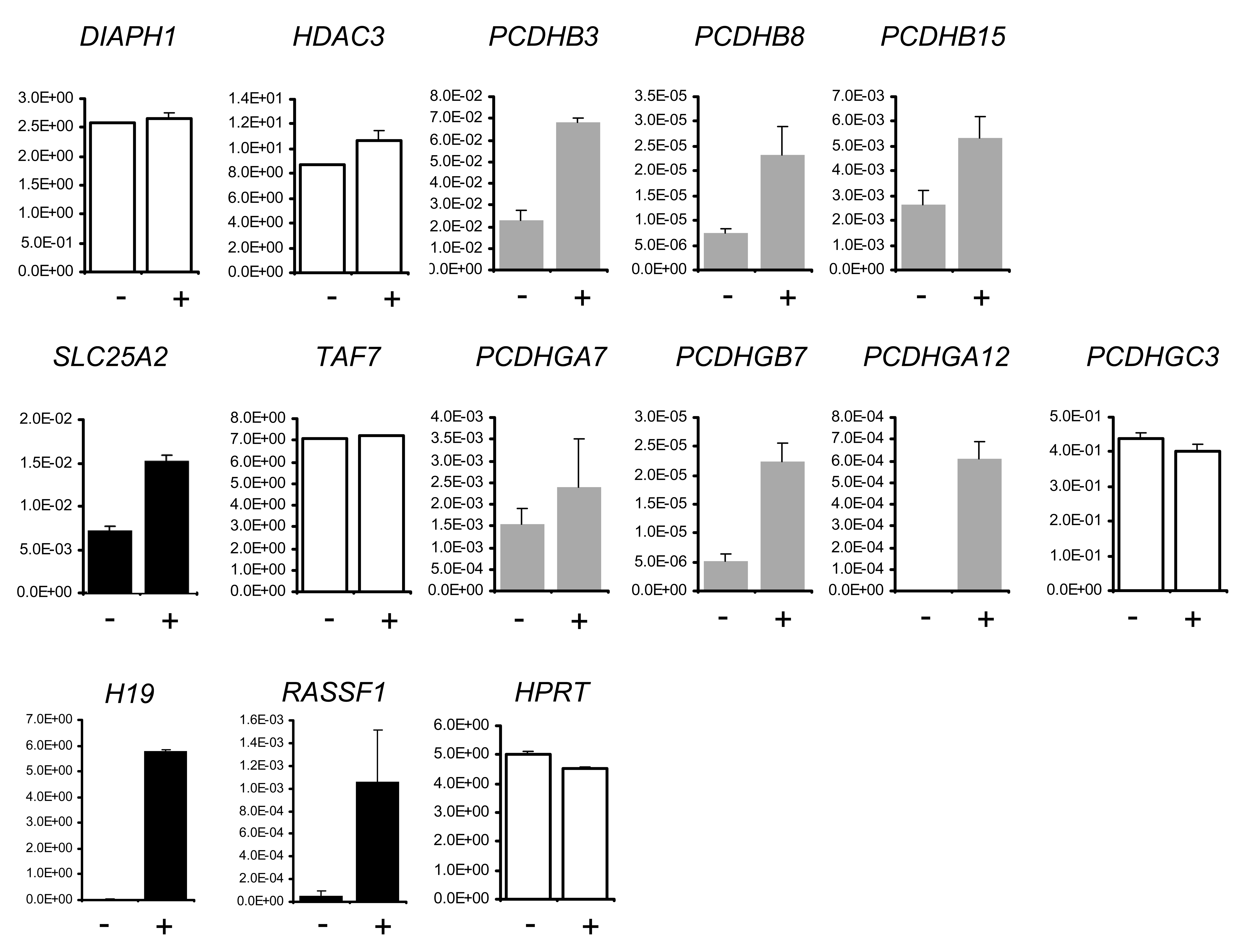

Supplement: Figure S5 — Pharmacological demethylation of WiT49 cells. Quantitative real-time RT-PCR of 5q31 transcripts, mock-treated (-) or 5-azacytidine treated (+) cells. Grey bars indicate genes associated with hypermethylated CGIs, white bars represent genes with CpG islands with no detectable methylation and black bars are used for SLC25A2. HPRT is an X-chromosome housekeeping control gene. DIAPH1 and HDAC3 are located on chromosome 5q31 outside the LRES. Expression data for 3 PCDHB@ genes and 4 PCDHG@ genes is shown relative to TBP, together with SLC25A2 and TAF7 genes, which are located within the LRES. Induction of the WT hypermethylated control genes RASSF1 and H19 is also shown. (2.41 MB TIF) [file pgen.1000745.s005.tif]

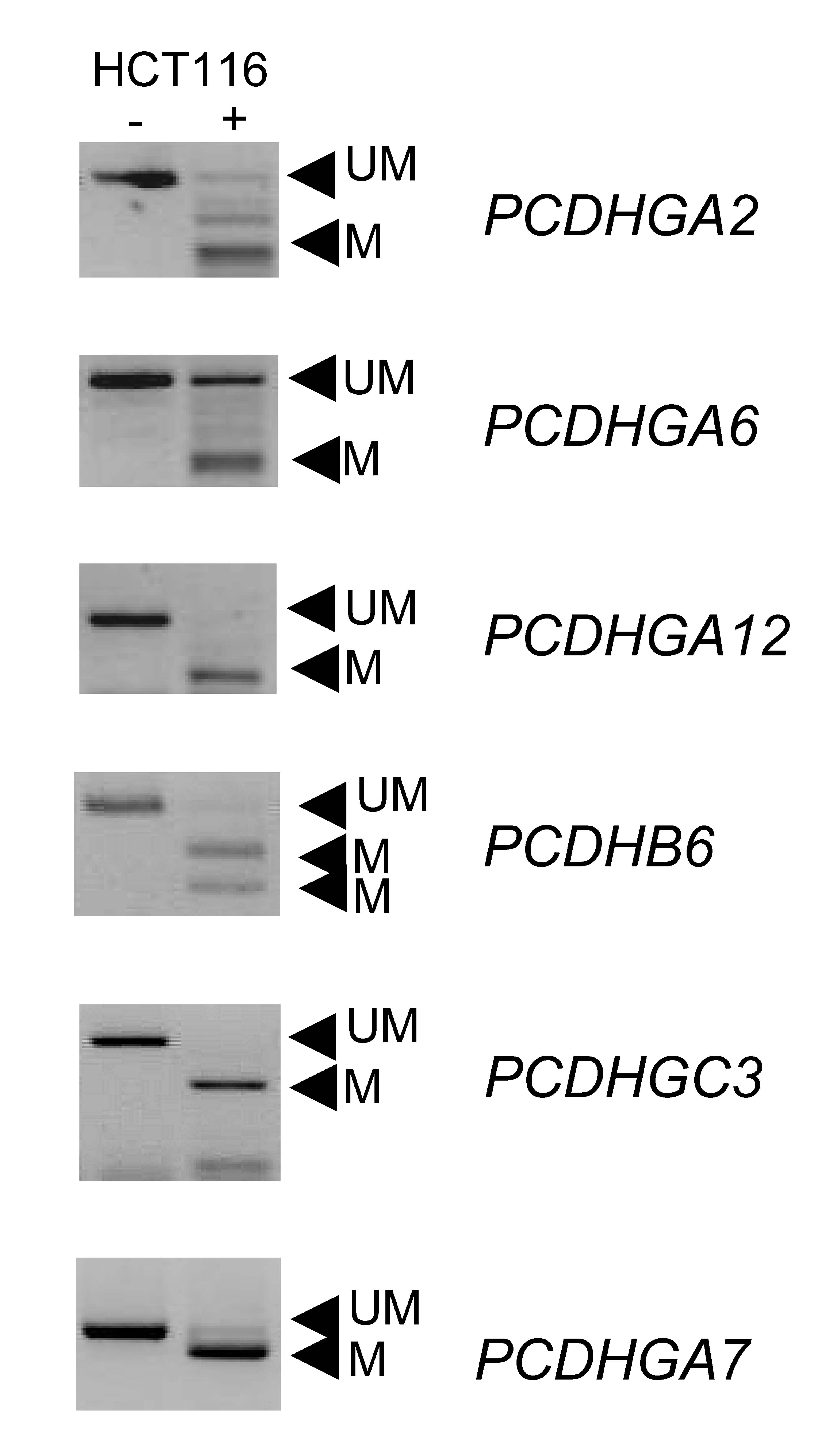

Supplement: Figure S6 — Hypermethylation of PCDHGA2, PCDHGA6, PCDHGA12, PCDHB6, PCDHGC3 and PCDHGA7 in HCT116 cells demonstrated using COBRA analysis. Arrowheads show methylated (M) and unmethylated (UM) DNA fragments; presence or absence of restriction enzyme is indicated (+/−). (1.25 MB TIF) [file pgen.1000745.s006.tif]

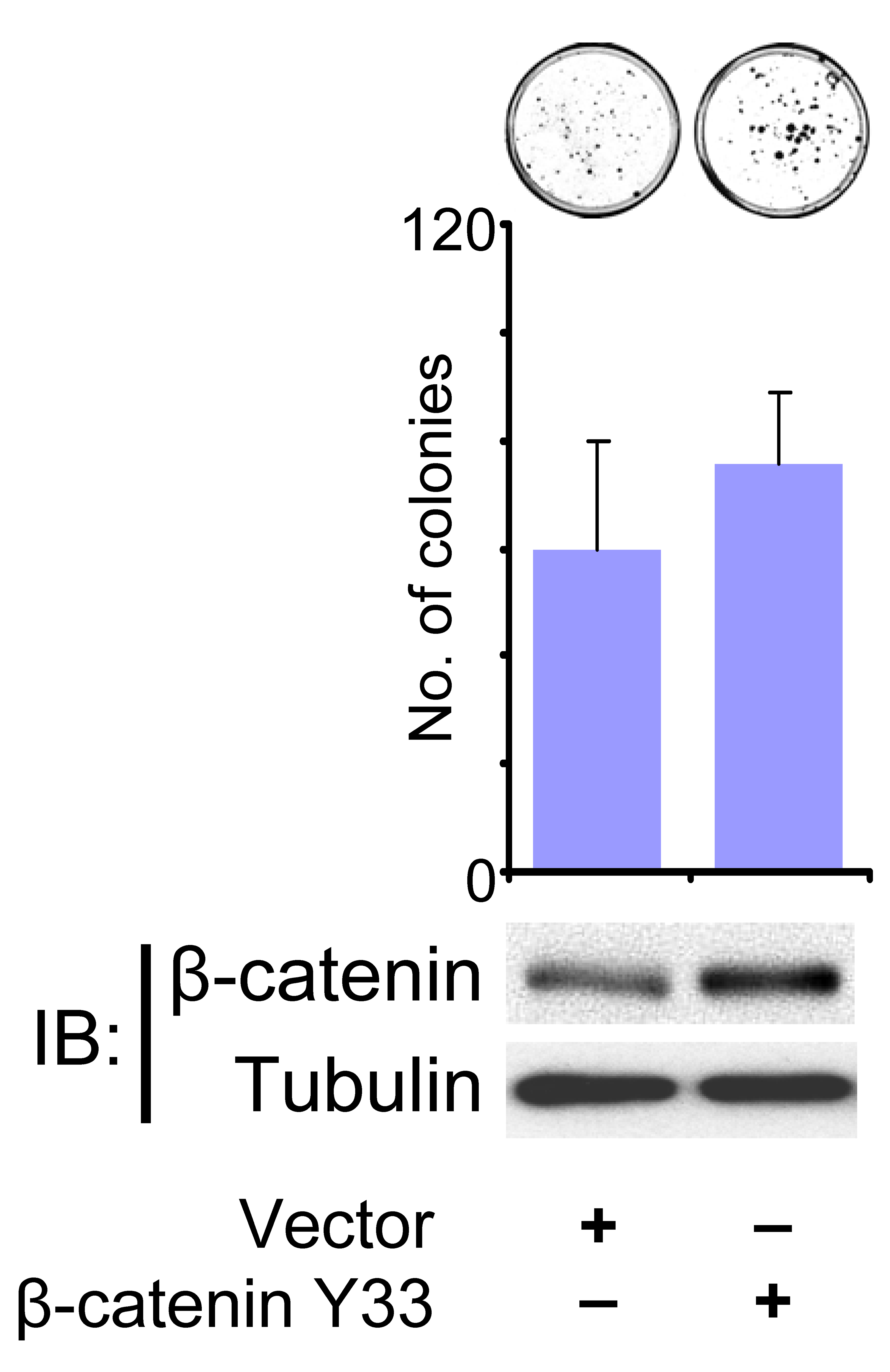

Supplement: Figure S7 — Suppression of colony formation is not dependent on non-specific toxicity of transfected genes. Mutant β-catenin (Y33, tyrosine at amino-acid 33) expression does not suppress colony formation in HEK293 cells. HEK293 cells were transfected with CTNNB1 cDNA cloned in the same expression vector (pcDNA3.1/Zeo) as PCDH constructs. After selection and staining, plates were photographed and colony counts determined for each transfection. Representative plates (above) and mean colony counts (below) are shown. Verification of β-catenin protein expression after transfection is shown by immunoblotting below the histograms, together with tubulin to control loading. (1.35 MB TIF) [file pgen.1000745.s007.tif]

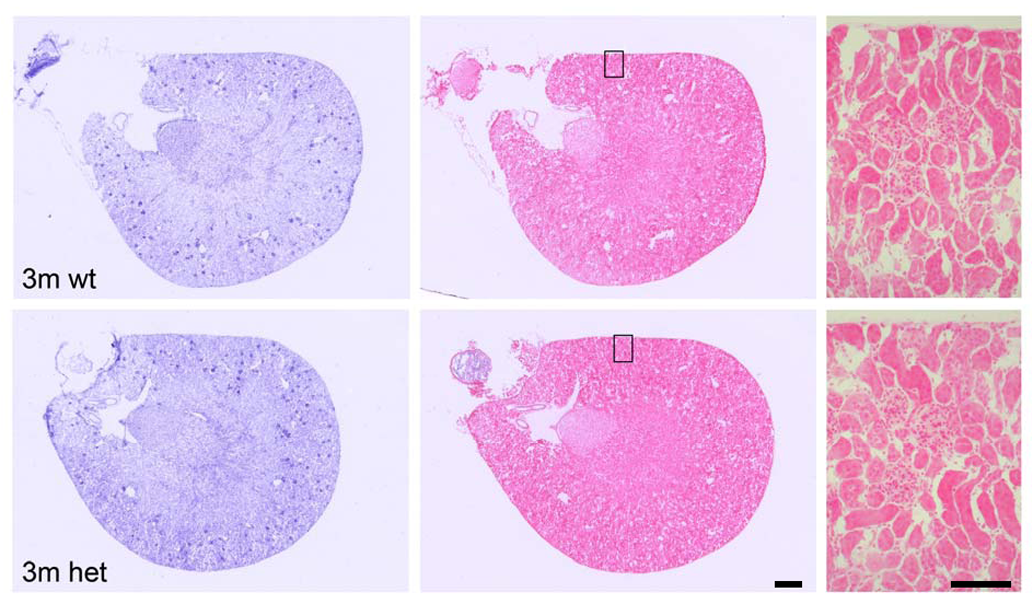

Supplement: Figure S8 — Kidneys of heterozygous Pcdhg@ mutant mice show no malformations (see Text S1). Histology of three-month old wild-type (wt, n = 2) and heterozygous Pcdhg@ mutant kidneys (het, n = 3) was examined on cryosections. Staining of adjacent sections with cresyl-violet (left column) and nuclear fast red (middle and right columns) was used to highlight the cytoarchitecture of the specimens. The overall morphology of the heterozygous kidneys appeared normal and showed no malformations. Scale bars = 500 µm. At higher magnifications, findings were comparable in three-month old wild-type and heterozygous littermates and displayed normal cytoarchitecture in aged heterozygous mice (boxed areas are shown in the right column, scale bars = 100 µm). (1.02 MB TIF) [file pgen.1000745.s008.tif]
