# Supplementary material for: Frequent Long-Range Epigenetic Silencing of Protocadherin Gene Clusters on Chromosome 5q31 in Wilms' Tumor
Source: PLoS Genet. 2009 Nov 26;5(11):e1000745. doi: 10.1371/journal.pgen.1000745 (PMC2776977; doi:10.1371/journal.pgen.1000745)
Supplement: Text S1 — Supporting information methods. (0.03 MB DOC) [file pgen.1000745.s013.doc]

**Text S1**

Supporting information Methods:

Kidneys of heterozygous *Pcdhg@* mutant mice and wild-type littermates were from mixed background black 6 mice. Adjacent cryosections of fresh frozen tissues (14 µm) were collected on Superfrost Plus slides (Menzel, Germany) and stained with 0.5% cresyl-violet-acetate in water (Sigma). Alternatively, sections were fixed and processed for β-galactosidase reactivity/LacZ-staining for 20 hours and after washing were counterstained with nuclear fast red solution (Roth, Germany). The *Pcdhg@* mutant allele was generated from mouse embryonic stem cells (ES cells) harbouring a gene-trap lacZ insertion between exons 2 and 3 in the *Pcdhg@* constant region (obtained from the German Gene Trap Consortium. http://genetrap.gsf.de; ES cell clone A030A06) as described elsewhere (M. Ebert et al., manuscript in preparation, see also Ebert M., Kemler R. and M. Frank (2005) Disruption of the Protocadherin-Gamma Cluster in Gene-trap Mice Leads to Neuronal Degeneration and Perinatal Lethality. *Proceedings of the 6^th^ Meeting of the German Neuroscience Society / 30th Göttingen Neurobiology Conference*, Neuroforum 11(1), supplements, p.1038, <http://www.neuroanatomie.uni-goettingen.de/neurobio_archiv/2005/pdf/Proceedings-Goettingen2005.pdf>). The mutant *Pcdhg@* allele gives rise to Pcdhg-lacZ fusion proteins lacking most of the C-terminal intracellular domain.
